# Tuning BMP-Regulated Cell Differentiation in the Aortic Media by Mutating Matrix Gla Protein

**DOI:** 10.1161/ATVBAHA.126.324621

**Published:** 2026-07-16

**Authors:** Xinjiang Cai, Kavinda K.J. Gunasinghe, Lei Qi, Li Zhang, Xiuju Wu, Zheng Jing, Yan Zhao, Hannah Kim, Eddy Yao, Tzung K. Hsiai, Taufiq Rahman, Xavier W. Chee, Yucheng Yao, Kristina I. Boström

**Affiliations:** 1 Division of Cardiology, Department of Medicine, David Geffen School of Medicine at the University of California, Los Angeles (X.C., L.Q., L.Z., X.W., Z.J., Y.Z., H.K., E.Y., T.K.H., Y.Y., K.I.B.).; 2 Faculty of Engineering, Computing and Science, Swinburne University of Technology Sarawak, Kuching, Malaysia (K.K.J.G., X.W.C.).; 3 Medicinal Chemistry, Monash Institute of Pharmaceutical Sciences, Monash University, Victoria, Australia (K.K.J.G.).; 4 Department of Bioengineering, Henry Samueli School of Engineering and Applied Science (T.K.H.), University of California Los Angeles 8783 .; 5 Molecular Biology Institute (K.I.B.), University of California Los Angeles 8783 .; 6 Division of Cardiology, Department of Medicine, Greater Los Angeles VA Healthcare System, CA (T.K.H.).; 7 Department of Pharmacology, University of Cambridge, United Kingdom (T.R.).; 8 Department of Biochemistry, Yong Loo Lin School of Medicine, National University of Singapore (X.W.C.).

**Keywords:** bone morphogenetic proteins, cell differentiation, fibrosis, matrix Gla protein, vascular calcification

## Abstract

**BACKGROUND:**

MGP (matrix Gla protein) serves as an inhibitor of vascular calcification by limiting elastin degradation and regulating BMP (bone morphogenetic protein) activity. Mutation of the conserved proline residue 64 to glycine in MGP abolishes BMP binding in vitro. We hypothesized that selective loss of BMP binding would elucidate the contribution of BMP to cell differentiation in *Mgp*-deficient mice.

**METHODS:**

Computational analyses using AlphaFold3 and molecular dynamics simulations were performed to determine the structural effects of γ-carboxylation and the proline residue 64 to glycine mutation. The vascular phenotype of *Mgp*-knockin mice expressing the proline residue 64 to glycine mutation was compared with wild-type (WT) and global *Mgp*-knockout mice. Proximity ligation assay was performed to assess MGP-BMP4 in aortic cells. Single-cell RNA-sequencing was used to identify cellular alterations in the vascular media.

**RESULTS:**

Molecular dynamics simulation revealed 5 Ca^2+^ ions coordinated by γ-carboxylated Glu residues in the MGP dimer. The proline residue 64 to glycine mutation did not disrupt Ca^2+^ binding but likely suppressed conformational changes required for BMP4 binding. Unlike *Mgp*-*KO* mice, *Mgp*-knockin mice did not develop vascular calcification, elastic lamina proteolysis, and endothelial-mesenchymal transition, but exhibited vascular fibrosis. MGP-BMP4 interaction observed in WT aortic cells was barely detectable in *Mgp*-knockin aortic cells. Single-cell RNA-sequencing revealed increased fractions of smooth muscle cells and enhanced myofibroblast differentiation in the *Mgp*-knockin aortas compared with WT. In *Mgp*-knockin aortas, SMAD2 expression was significantly increased throughout the vessel wall compared with WT aortas. In contrast, SMAD1/5/9 activation in the *Mgp*-knockout aortas was more confined to the endothelium, whereas it localized to the media-adventitia transition in WT and *Mgp*-knockin aortas. Both *Mgp*-knockin and *Mgp*-knockout mice developed arteriovenous malformations in the lungs, kidneys, and brain.

**CONCLUSIONS:**

Our findings suggest that MGP plays a versatile role in preserving vascular integrity, in part serving as an important BMP-trap aimed at directing vascular cell differentiation.

**What Are the Clinical Implications?**

Aortic fibrosis and the resulting stiffening are markers of vascular disease and predictors of cardiovascular events and mortality, driving outcomes such as stroke, heart failure, and hypertension. Although TGFβ (transforming growth factor β) signaling has been linked to fibrosis, the role of BMP (bone morphogenetic protein) signaling and its inhibitors, such as MGP (matrix Gla protein), is not known. This study suggests that BMP signaling is an upstream regulator of TGFβ signaling. Mutated MGP, incapable of binding BMP, allows activation of TGFβ signaling, which triggers myofibroblast activation and aortic fibrosis in mice. However, the loss of BMP binding does not cause aortic calcification. The results emphasize the importance of coordination between BMP and TGFβ signaling, requiring both to be functional and accurately regulated to limit fibrosis. Loss of BMP binding also leads to arteriovenous malformations in multiple organs, similar to the global MGP deletion. We conclude that BMP binding in MGP is critical for limiting aortic fibrosis and arteriovenous malformation formation, whereas MGP sequences not involved in BMP binding mediate the well-known anticalcific effects of MGP. The results are important for the design of therapies aimed at treating fibrosis versus calcification.

MGP (matrix Gla protein), a vitamin K–dependent extracellular matrix protein, was initially identified as an inhibitor of vascular calcification.^1–3^ Mice with global deletion of the *Mgp* gene (*Mgp*-knockout) exhibit profound arterial osteogenesis with progressive elasto-calcinosis that begins shortly after birth and contributes to premature death within 4 to 6 weeks.^3,4^ Several studies have shown that endothelial-mesenchymal transitions (EndMTs) driven by BMPs (bone morphogenetic proteins), the emergence of multipotent progenitor cells, proteolytic degradation of the elastic lamellae, and redifferentiation of smooth muscle cells (SMCs) contribute to the vascular calcification.^5–8^

MGP requires posttranslational modifications to attain a functional state. These modifications include phosphorylation of 3 N-terminal serine residues (Figure 1A), which provides elasto-protection, and γ-carboxylation of 3 (mouse) or 4 (human) glutamate residues located in the C-terminal Gla region, which participates in Ca^2+^ binding (Figure 1A).^9–12^ Elegant studies by Parashad et al^12^ showed that knockin mice with mutations in the 3 N-terminal serine residues closely replicate the mineralization phenotype of the global *Mgp*-knockout mice, although it was not clear whether the mutations affected cell differentiation. Studies from our lab showed that mutations of the conserved proline residue 64 (Pro64) to glycine (Pro64Gly) in MGP abolished the ability to bind and inhibit BMP4 in vitro, whereas the Pro64Phe mutation did not.^10^ In addition, at least 1 Ca^2+^-binding Gla-residue on each side of Pro64 was required to preserve the ability to bind BMP4.

**Figure 1.**
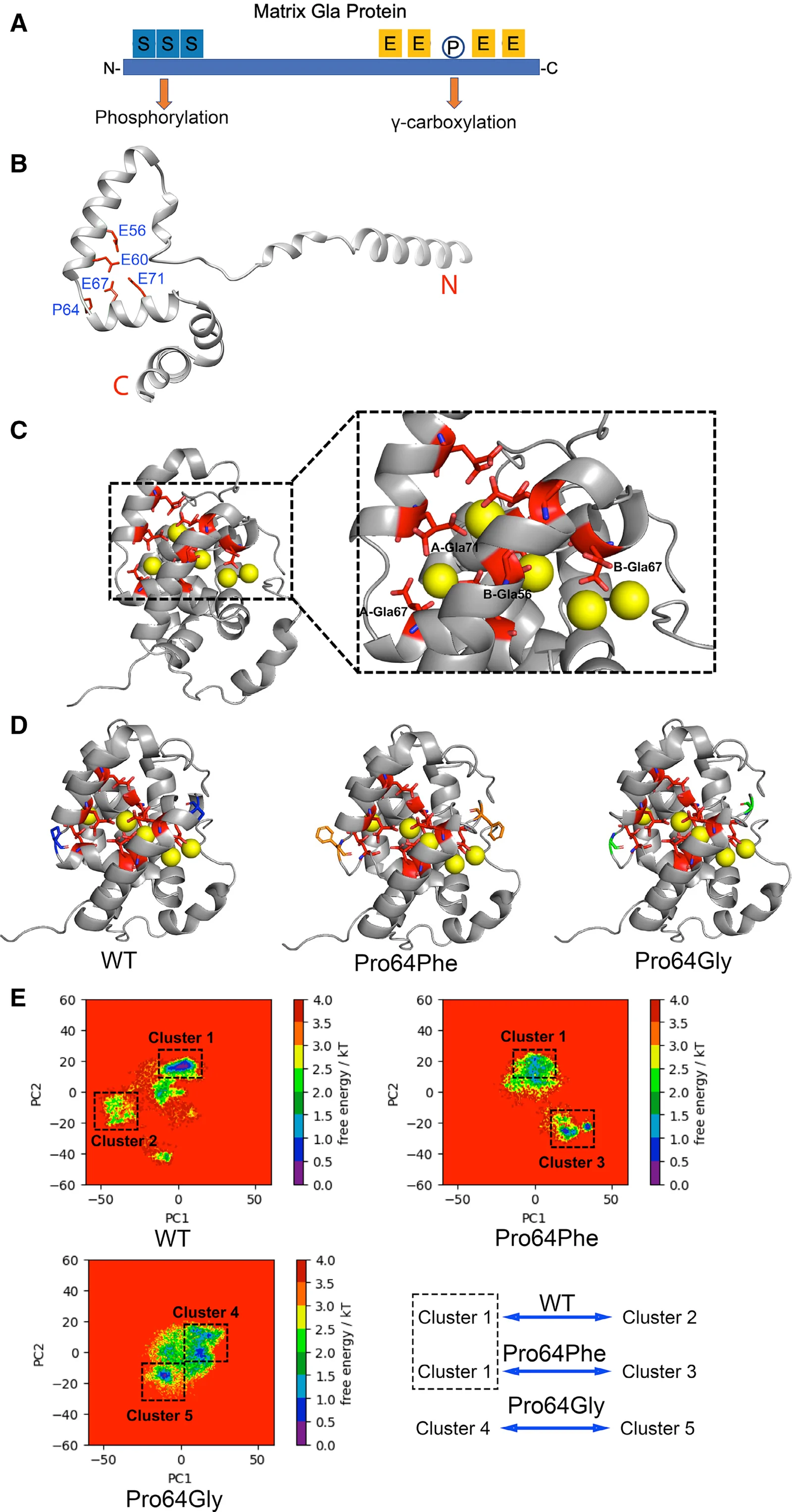
**Conformational properties of γ-carboxylated MGP (matrix Gla protein) predicted by AlphaFold and molecular dynamics simulations. A**, A schematic model of MGP depicting the positions of 3 N-terminal Ser residues that undergo phosphorylation and the proline residue 64 (Pro64) flanked on each side by 2 Glu residues subject to γ-carboxylation. **B**, AlphaFold3 structure of the MGP monomer illustrating the extended N-terminal helix and the C-terminal Gla-helix domain showing the locations of Pro64 and 4 Glu residues. **C**, Binding of 5 Ca^2+^ ions (yellow) to Gla67 and Gla71 in chain A and Gla56 and Gla67in chain B in the predicted MGP dimer, assessed by molecular dynamics simulations. Gla residues are shown in red. **D**, Conformational changes in MGP induced by mutating Pro64 to Phe or Gly. Pro64 is shown in blue, Phe64 in orange, and Gly64 in green. **E**, Free energy landscape plots from molecular dynamics simulations revealing a shared conformational basin between wild-type (WT) and Pro64Phe mutant in cluster 1, which is absent in the proline residue 64 to glycine (Pro64Gly) mutant. C indicates C-terminus; E, glutamate; N, N-terminus; P, proline; and S, serine

MGP also serves as a vascular morphogen in the development of the pulmonary and retinal vasculature through its ability to regulate BMPs.^11,13–18^ Loss of MGP triggers the development of arteriovenous malformations (AVMs),^15,17^ whereas excess of MGP mitigates AVMs caused by heterozygosity of *Alk1*^*+/*^^*−*^, the causative gene of hereditary hemorrhagic telangiectasia 2.^13^ BMP signaling is also upstream of TGFβ (transforming growth factor β) signaling as BMP activity induces *Alk5* (TGFβ type 1 receptor), through the induction of *Alk1*, *Alk2*, and activation of SMAD1/5/9 signaling.^15^

BMP signaling in the embryonic endothelium has been shown to be critical for early vascular coverage by mural cells.^19^ Enhanced BMP signaling results in increased numbers of *Acta2*-expressing SMCs and promotes a differentiated SMC phenotype. This effect may also occur through the loss of miR26a, which targets *Smad1*, or by forced *SMAD1* activation.^20^ A similar requirement for BMP signaling is evident in stem cell differentiation protocols for the SMC lineage, where a specific time period of BMP action is essential to achieve SMC lineage.^21,22^ In addition, TGFβ signaling initiated from the endothelial cells (ECs) is necessary for subsequent growth arrest and terminal differentiation of the SMCs.^23^

Despite evidence demonstrating that MGP regulates BMP activity, it remains unclear how this function operates separately from other MGP activities such as elastin protection mediated by the phosphorylated serines. In this study, we investigated the in vivo significance of MGP-regulated BMP activity in the aortic media using an integrated approach combining an *Mgp* Pro64Gly knockin mouse model, single-cell RNA-sequencing (scRNA-seq), and artificial intelligence–assisted protein structural analysis. We show that the Pro64Gly mutant retains the ability to protect against arterial calcification but increases BMP activation in the media-adventitia transition of the aorta. In addition, it enhances TGFβ signaling in the media and diverts cell trajectories towards myofibroblasts and SMCs, which ultimately results in aortic fibrosis. The enhanced BMP activation also causes the development of AVMs in multiple organs, including lungs, kidneys, and brain. Together, these findings help dissect the versatile functions of MGP as a vascular morphogen, demonstrating the importance of a regulatory BMP-trap in optimizing aortic cell composition and preserving vascular integrity.

## Material and Methods

### Data Availability

Data created for the study are available in a persistent repository.

The scRNA-seq data sets of wild-type (WT) and *Mgp*-knockin mice are accessible from the GEO (Gene Expression Omnibus) database with accession number GSE289219.

### Contacts for Reagent and Resource Sharing

Requests for further information, resources, and reagents should be directed to corresponding authors X.C. (xinjiangcai@mednet.ucla.edu) and K.I.B. (kbostrom@mednet.ucla.edu). Sharing of primary samples is based on availability and may be subject to Material Transfer Agreements and will require appropriate research ethics board certifications.

For Key Resources, see Table S1.

### Animals

A *Mgp*^mut/mut^ (*Mgp*-knockin) mouse, where mutation of Proline-64 to glycine eliminates BMP binding^10^ was generated by homologous recombination on C57BL/6J background as previously described.^18^ *Mgp*^*+/*^^*−*^ mice (B6.129S7-Mgptm1Kry/KbosJ, Stock No. 023811) was obtained from the Jackson Laboratory. Genotypes were confirmed by polymerase chain reaction (PCR).^3,18^ Male and female mice were exposed to a standard 12-hour:12-hour light/dark cycle and fed a standard laboratory diet (Diet 8604; Teklad Rodent Diet, Inotiv). Preliminary studies on 4-week-old mice did not detect significant differences between the sexes. Sex differences and interactions were not determined in this study. Equal number of male and female mice were included in each experimental group and analyzed together as indicated in the figure legends. Where indicated, male and female WT and *Mgp*-knockin mice were fed a high phosphorus diet (2% P & 1.2% Ca, Cat no. TD.120538, Inotiv) starting at 4 weeks of age and continued for 12 weeks. Both sexes were also included in these experiments; the results were similar, and mixed sex groups were analyzed together.

The studies were reviewed and approved by the institutional review board and conducted in accordance with the animal care guidelines set by the University of California, Los Angeles. The investigation also conformed to the standards of the National Research Council, *Guide* *for the Care and Use of Laboratory Animals*, *Eighth Edition* (Washington, DC; The National Academies Press, 2011).

### Computational Modeling of the MGP Homodimer with Ca^2+^

The MGP homodimer was modeled using AlphaFold 3 by employing the UniProt sequence for MGP (UniProt ID: H0YGZ6) and 3 Ca2+ ions.^24^ The regions in each monomer, spanning from Glu27 to Arg97, which are important for Ca2+ binding, exhibited high confidence with a predicted long-distance difference test >70. Subsequently, an additional 3 Ca^2+^ ions were introduced to the cavity containing Glu56, Glu60, Glu67, and Glu71 in the MGP dimer in both monomers to evaluate Ca^2+^ interactions with MGP. The aforementioned Glu residues in both monomers of MGP were modified into γ-carboxyglutamic acid (Gla) residues using the CHARMM-GUI server.^25^ The Pro64Phe and Pro64Gly mutations were added using PyMOL. All molecular dynamics (MD) simulations were performed using the AMBER 20 MD Simulation Package.^26^ The MD simulations were performed in 2 stages: the first stage of simulations to evaluate the Ca^2+^ uptake by MGP and the second stage to evaluate the effects of Pro64Phe and Pro64Gly mutations in MGP. The Gla modifications were parameterized using the AMBER GAFF force field while standard protein residues were parameterized using the ff14SB force field.^26^ The excess charges were neutralized with Cl-counter ions, and the system was solvated using TIP3P water molecules.^26^

Minimization was performed in 2 phases for each replicate. The first phase consisted of 5000 steps of the steepest descent, followed by 5000 conjugate gradient minimizations. Both restraint force and PME cutoff distance were kept at 300 kcal/mol and 10 Å, respectively. The second phase consisted of 10 000 steps of steepest descent and 10 000 conjugate gradient minimization steps with a PME cutoff distance of 10 Å without any restraints. Next, heating was performed for 200 ps, gradually increasing the temperature from 0 K to 300 K with the NVT ensemble, which was followed by 5 ns of equilibration with the NPT ensemble while production was performed for 500 ns for each replicate. Temperature and pressure were kept at 300 K and 1.0 bar, respectively, during equilibration and production stages and monitored using the Langevin thermostat and Berendsen barostat, respectively. For the second stage, the most stable conformation of MGP from the first stage’s triplicate simulations was used. The WT MGP, along with the Pro64Phe and Pro64Gly variants, were subjected to similar parameterization, minimization, heating, equilibration, and production as outlined in the first stage of MD simulations and simulated in triplicate.

All MD trajectories were evaluated using the first frame as reference using cpptraj, pytraj, and PyEMMA modules.^27,28^ The Free Energy Landscapes and the Markov State Models for transition state probabilities were calculated using the combined triplicate simulations for WT MGP and the Pro64Phe and Pro64Gly variants with 12 500 frames from each simulation run.

### Immunofluorescence and Quantification

Immediately after euthanasia with isoflurane, mice were transcardially perfused with 50 mL of 1× PBS per mouse. Tissues were then harvested and fixed in 4% paraformaldehyde overnight at 4 °C. After 2 washes with 1× PBS, the tissues were dehydrated in 70% ethanol and embedded in paraffin. Serial 5-µm sections were obtained from the paraffin blocks, followed by deparaffinization and rehydration. Antigen retrieval was performed using a citrate-based unmasking solution (H-3300, Vector Laboratories).

Immunofluorescence was performed as previously described.^14,29^ Tissue sections were permeabilized for 30 minutes using 0.2% Triton X-100 and then blocked with 5% chicken serum in PBS at room temperature for 1 hour. The specimens were then incubated with primary antibodies overnight at 4 °C. See Table S1 for antibody information. The following primary antibodies were used: CD31 (R&D Systems), MGP (Abcam), α-SMA (α-smooth muscle actin; R&D Systems), phospho-SMAD1/5/8 (Cell Signaling), phospho-SMAD2 (Cell Signaling); VWF (von Willebrand Factor; Dako). Alexa Fluor–conjugated secondary antibodies (Invitrogen; 1:500) were applied, and the sections were costained with DAPI (4',6-diamidino-2-phenylindole; 1:5000, Sigma-Aldrich). Negative controls included staining with nonimmune IgG isotype antibodies and staining without primary antibodies. Nonmineralized tissue from *Mgp*-knockout mice served as a negative control for anti-MGP antibodies. Image acquisition was performed with an inverted Nikon Eclipse Ti–S microscope (Nikon Corporation) or an All-in-One Fluorescence Microscope BZ-X800 (Keyence). ImageJ software (version 1.54) was used to perform semiquantitative analysis of immunofluorescence staining.

### Isolation and Treatment of Aortic Cells

Aortic cells were isolated from WT and *Mgp*-knockin mice and cultured for 24 hours before treatment. Cells were treated with recombinant BMP4 (50 ng/mL; R&D Systems), SB431542 (5 μmol/L; Selleck Chemicals), or recombinant TGFβ1 (5 ng/mL; R&D Systems) for 48 hours and then were harvested for total RNA extraction and quantitative reverse transcription PCR analysis.

### Proximity Ligation Assay

The proximity ligation assay was performed on isolated aortic cells obtained from WT and *Mgp*-knockin mice using the Duolink in situ Detection Reagents Red kit (Sigma-Aldrich). MGP (Proteintech) and BMP4 (Abcam) primary antibodies were applied according to the manufacturer’s instructions. Nuclei were counterstained with DAPI. Fluorescent proximity ligation assay signal images were obtained using fluorescence microscopy, and images were processed with ImageJ software for quantification as previously described.^30^

### Serum TGFβ1 and BMP4 Quantification

Mouse serum TGFβ1and BMP4levels were quantified using the Mouse TGFβ1 DuoSet ELISA kit (Biotechne, R&D Systems) and the Mouse BMP4 ELISA Kit (Antibodies.com LLC), respectively, according to the manufacturers’ instructions.

### In Vivo Microcomputed Tomography Imaging of Aortic Calcification

Microcomputed tomography imaging was performed at the Crump Molecular Imaging Technology Center at UCLA, as previously described.^31^ Briefly, mice were anesthetized with isoflurane and scanned with a high-resolution, volumetric microcomputed tomography scanner (CrumpCAT, designed and built by the Crump Imaging Center at UCLA^32^). The image data were acquired with a 10 µm isotropic voxel resolution, 200 ms exposure time, 2000 views, and 5 frames per view. The resulting DICOM files were analyzed to create volume renderings of the regions of interest. Raw data files were viewed using MicroView 3-dimensional volume viewer & analysis tool (GE Healthcare) and Osirix software (Pixmeo SARL).

### Vascular Shunting to Assess AVM

Immediately after the mice were euthanized, 15 µm GFP (green fluorescent protein)–labeled fluorescent microspheres (FluoSpheres, Invitrogen) were injected into the left ventricle. The organs were then examined and photographed under fluorescence microscopy.

### Mouse Echocardiography

Sizes of the mouse aorta were measured using the Vevo 3100 imaging system (FUJIFILM VisualSonics). Briefly, the mice were anesthetized with 1.5% isoflurane in 95% oxygen. Body temperature was maintained at 37° on a heating pad. Continuous ECG was used to monitor heart rates, which were maintained between 400 and 500 bpm. A right-side superior parasternal view was utilized to acquire a 2-dimensional image plane containing the ascending aorta, aortic arch, and proximal portion of the descending aorta in 1 view. Aortic sinus, sinotubular junction, and middle ascending aorta were measured at diastole.

### Transmission Electron Microscopy

Transmission electron microscopy experiments were performed as previously described.^5^ Briefly, dissected aortic tissues were fixed in 2% glutaraldehyde and 2% paraformaldehyde in 0.1 mol/L PBS (pH 7.4) for 2 hours at room temperature and then incubated overnight at 4 °C. The next day, tissues were treated with 0.5% tannic acid for 1 hour, washed 5× in 0.1 mol/L PBS, and postfixed in 1% osmium tetroxide in PBS. After washing 4× in Na acetate buffer (pH 5.5), samples were block-stained in 0.5% uranyl acetate in 0.1 mol/L Na acetate buffer, pH 5.5, for 12 hours at 4 °C. Dehydration was performed in graded ethanol (50%, 75%, 95%, 100%, 100%, 100%) for 10 minutes each. The samples were then passed through propylene oxide and infiltrated in mixtures of Epon 812 and propylene oxide 1:1 and then 2:1 for 2 hours each, followed by infiltration with pure Epon 812 overnight. Embedding was completed in pure Epon 812, cured at 60 °C for 48 hours. Ultrathin sections (60 nm, gray interference color) were cut using a diamond knife on an RMC MTX ultramicrotome. Sections were mounted on single-hole grids coated with Formvar and carbon, then double-stained with 8% uranyl acetate for 25 minutes at 60 °C and lead citrate for 3 minutes at room temperature. The sections were subsequently examined using a JEOL 100CX electron microscope.

### Quantitative Reverse Transcription PCR

Total RNA was extracted from tissues using RNeasy Mini kits (Qiagen). cDNA was synthesized using the high-capacity cDNA reverse transcription kit (Thermo Fisher Scientific) according to the manufacturer’s instructions. Quantitative reverse transcription PCR was performed on a 7500 Fast Real-Time PCR System (Applied Biosystems) with TaqMan Universal PCR Master Mix (Thermo Fisher Scientific). The cycling conditions were as follows: 50 °C for 2 minutes, 95 °C for 10 minutes, followed by 40 cycles at 95 °C for 15 s and 60 °C for 1 minute. Threshold cycle values of specific cDNAs were normalized to the housekeeping gene glyceraldehyde-3-phosphate dehydrogenase (*Gapdh*) and expressed as relative values. See Table S2 for primers used in quantitative reverse transcription PCR.

### Cell Isolation, Single-Cell RNA-Sequencing Analysis, and Pseudotime Trajectory Construction

Aortic cell isolation was performed with both male and female WT and MGP-knockin mice at 1 month old as previously described.^33^ After the mice were sacrificed and perfused with 10 mL of warm 1× PBS without Ca^2+^ and Mg^2+^, aortas were isolated, and adventitia were removed under a dissecting microscope. The dissected aortas were then cut into small pieces and incubated with 1 mL of digestion buffer containing DNase1 (Qiagen), 10 mmol/L HEPES, Liberase (Millipore-Sigma), and 1× Hanks’ balanced salt solution at 37 °C for 25 minutes. Isolated cells in a single cell suspension were washed 3× and passed through a 40 µm cell strainer (Corning). The final cell suspension was resuspended in 1×PBS containing 1% fetal bovine serum and 0.05% bovine serum albumin. Dead cells, identified by DAPI positivity (1:5000; Sigma-Aldrich), were removed by fluorescence-activated cell sorter. The remaining live cells were sent for scRNA-seq at the UCLA Technology Center for Genomics & Bioinformatics.

ScRNA-seq data were obtained and analyzed as described previously.^29^ ScRNA-seq libraries were generated using the Chromium Single Cell 3′ v3 assay (10x Genomics). The libraries were sequenced on the Illumina NextSeq 6000 platform with a depth of ≈300 million reads per library. Raw base call files generated from paired-end sequencing were first demultiplexed and converted to FASTQ format using the CellRanger mkfastq function (v7.1.0; 10x Genomics). Sequencing read quality was assessed using standard quality control metrics, including read quality scores, sequencing depth, and barcode performance. High-confidence reads were subsequently processed using the CellRanger count pipeline (v7.1.0). Briefly, reads were aligned to the mouse reference genome (mm10) using the STAR aligner implemented within CellRanger. The CellRanger count function performed read alignment, assignment of reads to cell barcodes, and unique molecular identifiers, and quantification of gene expression to generate feature (gene)-barcode matrices using default parameters. Cells and genes passing CellRanger default filtering criteria were retained for downstream analyses.

The R package Seurat (v5) was used for data processing, cell clustering, and differentially expressed gene visualization.^34^ Cells were first quality filtered to have >200 detected genes, <15 000 RNA counts, and 10% of mitochondrial genes. Data were transformed, and mitochondrial genes were regressed out. Data from WT and *Mgp*-knockin aortas were integrated, and dimensional reduction, cell clustering, and data visualization were performed using Uniform manifold approximation and projection dimensional reduction. Pseudotime trajectories in a subset of data were constructed using the R package Monocle 3 (v2.18.0).^35^

### Statistical Analysis

Statistical analyses were performed using GraphPad InStat (version 9.0; GraphPad Software Inc). Data were represented as mean±SEM. Data normality was assessed using the Shapiro-Wilk test and homogeneity of variance was evaluated using the *F* test or Bartlett test. Unpaired 2-tailed Student *t* test for 2-group comparisons and 1-way ANOVA with Tukey multiple comparisons tests for multiple-group comparisons when both assumptions were satisfied. For multiple-group comparisons, when normality was satisfied but homogeneity of variance was not, Welch ANOVA was applied. *P* values of <0.05 were considered statistically significant, and experiments were repeated a minimum of 3×.

## Results

### MD Simulations Reveal That Gla Residues Mediate Ca^2+^ Binding in MGP

γ-Carboxyglutamic acid (Gla) residue–dependent Ca^2+^ binding in MGP and osteocalcin is believed to induce α-helical conformational changes to increase their affinity for hydroxyapatite.^36^ The crystal structure of MGP has not been determined partly due to its highly insoluble nature,^36^ but the dimeric crystal structure of osteocalcin in binding to hydroxyapatite has been resolved.^37^ The Gla-helix domain of MGP shares significant homology with that of osteocalcin, and both MGP and osteocalcin contain a highly conserved proline residue in the Gla-helix domain, flanked by Ca^2+^-binding Gla residues.^37^

To further investigate the role of γ-carboxylation in MGP’s Ca^2+^ binding capability and whether the conformational changes caused by mutating Pro64 at the atomic level affect the structural landscape in WT versus MGP mutants, we conducted MD simulations on the predicted protein structure of human MGP from AlphaFold3^24^ with high-confidence scores in the Gla-helix domain when compared with the osteocalcin structure. We focused on the effect of the Pro64Gly and Pro64Phe mutations on the properties that allowed for Ca^2+^ and BMP binding.

The human MGP structure predicted by AlphaFold3 contains an extended N-terminal strand and a C-terminal Gla-helix domain comprising 3 α-helices (Figure 1B). Pro64 is located between α1 and α2 helices, surrounded by 2 Glu residues on each helix (Glu 56 and 60, and Glu 67 and 71, respectively). Because MGP is known to be present as a dimer,^10^ we first applied 3 Ca^2+^ ions embedded in the MGP dimer and increased the total number of Ca^2+^ ions to 6 in the Gla-helix domains (Figure S1A). We then subjected the 4 Glu residues (Glu 56, 60, 67, and 71) to γ-carboxylation using CHARMM-GUI and performed MD simulations for 500 ns in triplicate to assess the optimal number of Ca^2+^ ions that bind to the MGP homodimer (Figure S1B). The combined free energy landscape plot of the triplicate MD simulations indicated that 5 Ca^2+^ ions bind to the 4 Gla residues in the MGP Gla-helix domain (Figure 1C), consistent with the spatial coordination of 5 Ca^2+^ ions in the osteocalcin dimeric structure.^37^ After calculating the chelation distance between each Ca^2+^ ion with their potential binding Gla residues in the Gla-helix domain, we identified that Gla67 and Gla71 in chain A and Gla56 and Gla67 in chain B were coordinated in the most stable conformational status with the average chelation distance <3 Å (Figure S1C).

Our previous studies suggested that the Pro64Gly mutation did not affect Ca^2+^ binding by MGP.^10^ In addition, the Pro64Gly mutation, but not the Pro64Phe mutation, loses the capability to bind BMP4.^10^ In Figure 1D and Figure S1D, we modeled the Pro64Phe and Pro64Gly mutants and compared their conformational changes in response to γ-carboxylation and Ca^2+^ binding affinity with the WT MGP. Modeling results indicated that MGP WT and the 2 mutants bind to Ca^2+^ in a similar fashion; however, the 3 Free Energy Landscape clusters suggested that both WT and the Pro64Phe mutant, but not the Pro64Gly mutant, share a common cluster 1 (Figure 1E). We speculate that cluster 1 may represent the essential conformational features required for MGP to bind to BMP4, and these features are conserved in the Pro64Phe mutant but absent in the Pro64Gly mutant. Together, this supports that Ca^2+^-binding would not be expected to change by the Pro64Gly mutation, but that conformational changes allowing for BMP4 interactions would be suppressed.

### Pro64Gly Mutation in *Mgp*-Knockin Mice Does Not Induce Aortic Calcification

**Figure 2.**
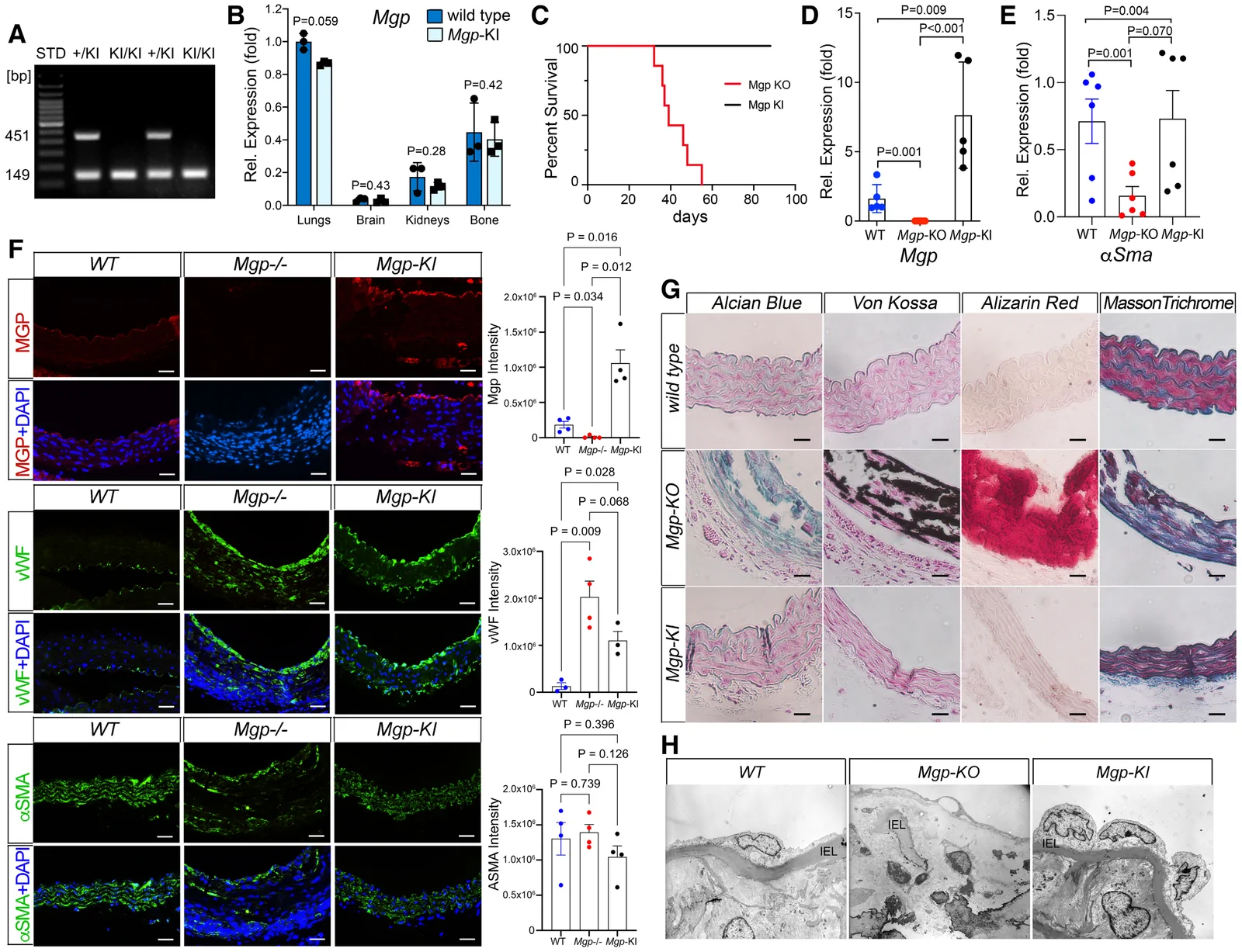
***Mgp* (matrix Gla protein)–knockin (KI) mouse does not exhibit vascular calcification phenotype. A**, Genotyping showing *Mgp* wild-type (WT; +) band of 451 bp and *Mgp*-KI band of 149 bp. **B**, Comparison of *Mgp* expression in organs from WT and *Mgp*-KI mice at 4 weeks of age, as determined by quantitative polymerase chain reaction (qPCR; n=3 mice per group; 2 males, 1 female). The *Mgp*-KI mice expressed the mutated proline residue 64 to glycine (Pro64Gly)-MGP. Unpaired 2-tailed Student *t* tests. **C**, Survival plot of *Mgp*-KI mice compared with *Mgp*-knockout (KO) mice, 8 mice per group (4 males, 4 females) over 90 days. **D**, Aortic expression of *Mgp*, and **E**, *Asma* (**right**) in WT, *Mgp*-KO, and *Mgp*-KI mice at 4 weeks of age, as determined by qPCR (n=6 mice per group; 3 males, 3 female). One-way ANOVA with Tukey post hoc test. **F**, Immunofluorescence for MGP (red, **top**), vWF (von Willebrand Factor; green, **middle**) and α-SMA (α-smooth muscle actin; green, **bottom**) of aortas from WT, *Mgp*-KO and *Mgp*-KI mice, at 4 weeks of age. DAPI (4',6-diamidino-2-phenylindole) was used to visualize nuclei. Scale bars, 20 µm. Intensity was determined by ImageJ (n=3 sections from male mice; 4 fields of view per section). One-way ANOVA with Tukey post hoc test. **G**, Histochemical staining (Alcian Blue, Von Kossa, Alizarin Red, and Masson Trichrome) of aorta from WT, *Mgp*-KO and *Mgp*-KI mice at 4 weeks of age. Scale bars, 20 µm. **H**, Transmission electron microscopy of the endothelium from WT, *Mgp*-KO and *Mgp*-KI aortas at 4 weeks of age. Magnification for EM, 3.7×10^3^. **G** and **H** each are representative of 3 independent experiments (sections from 2 males, 1 female). EM indicates electron microscope; IEL, internal elastic lamina; Rel., relative; and STD, DNA standards.

To investigate the in vivo significance of the disruption in BMP binding caused by the Pro64Gly mutation in MGP, we took advantage of the *Mgp*-knockin mouse model.^18^ The *Mgp*-knockin mouse model was generated by homologous recombination to express the Pro64Gly mutated form of MGP, which was verified by genomic DNA and cDNA sequencing^18^ and PCR genotyping (Figure 2A). The expression level of the *Mgp*-Pro64Gly mutant in *Mgp*-knockin mice was comparable to that of endogenous MGP in WT mice in lungs, brain, kidneys, and bone, as determined by quantitative PCR (qPCR) (Figure 2B). Compared with the global *Mgp*-knockout mice, which die prematurely between 4 and 8 weeks of age due to aortic calcification and rupture,^3^ the *Mgp*-knockin mice showed no premature death and survived to the end of the observation period (3–4 months; Figure 2C), suggesting less severe pathology.

In the aorta, qPCR showed the expression of the mutated form of *Mgp* was about 6-fold higher compared with WT *Mgp* by qPCR (Figure 2D), and *α-SMA* expression was reduced in the *Mgp*-knockout but not in *Mgp*-knockin aortas when compared with WT (Figure 2E). Immunofluorescence using anti-MGP antibodies detected MGP staining throughout the aortic wall, with the strongest staining in proximity to the intimal endothelium (Figure 2F). No MGP was detected in the *Mgp*-knockout aortas. The endothelium was labeled by VWF staining in WT, *Mgp*-knockout, and *Mgp*-knockin aortas (Figure 2F). However, vWF staining was more prominent in the vascular media of *Mgp*-knockout aortas, where previous studies have shown EndMTs,^5,6^, compared with *Mgp*-knockin aortas. Furthermore, immunofluorescence for αSMA, a marker of SMCs and mural cells, was detected at comparable levels in all aortas (Figure 2F), although the staining was dispersed in the intimal and adventitial sides of *Mgp*-knockout aortas compared with WT and *Mgp*-knockin aortas.

As previously reported, mineralization of the aorta and other elastic arteries was detected in the global *Mgp*-knockout mice within the first week of life and progressed to involve the entire aorta at about 4 weeks of age.^3,4^ However, mineralization was absent in the *Mgp*-knockin mice at the same age, as shown by Von Kossa and Alizarin Red S staining (Figure 2G; Figure S2A). Similarly, cartilaginous changes were absent in the *Mgp*-knockin aortas but detected in *Mgp*-knockout aortas by Alcian Blue staining (Figure 2G; Figure S2A). In contrast, fibrotic changes with increased collagen deposition, as shown by Masson Trichrome staining, were detected in both the *Mgp*-knockout and *Mgp*-knockin aortas (Figure 2G; Figure S2A). We also observed that the *Mgp*-knockin aorta frequently showed reduced spacing between the elastic lamellae and decreased curvature in the elastic lamellae compared with WT.

Global *Mgp* deletion triggers protease induction, EndMTs, and the emergence of abnormal ECs that contribute osteogenic progenitor cells to calcific lesions.^5,6^ Other investigators found the *Mgp* deletion similarly triggers protease activity in the media associated with vascular calcification.^8^ However, in *Mgp*-knockin aortas, we detected no significant induction of the proteolytic enzymes *Klk1*, *Klk5*, *Klk6*, *Ela1*, *Ela2*, the EndMT markers *Cd90*, *Cd44*, *Cdh2*, *Sox2*, and *Oct4*, or the osteogenic marker *Runx2* compared with WT aortas, although their expression levels were markedly elevated in *Mgp*-knockout aortas (Figure S2B and S2C). We also examined structural changes in the mouse aortas using transmission electron microscopy. The internal elastic lamina and the endothelium were severely disrupted in the *Mgp*-knockout aortas (Figure 2H) as previously shown.^6^ In contrast, the internal elastic lamina appeared intact in the *Mgp*-knockin aorta but with ECs that exhibited a less elongated shape compared with the morphology observed in the WT aortas. The results suggest that the Pro64Gly MGP still protects against proteolytic destruction, EndMTs, and osteogenesis in the aorta. In addition, direct measurement of aortic size by echocardiogram indicated comparable size between WT and *Mgp*-knockin aorta (Figure S2D).

### TGFβ Signaling Is Activated in Both Mgp-Knockout and Mgp-Knockin Mice

**Figure 3.**
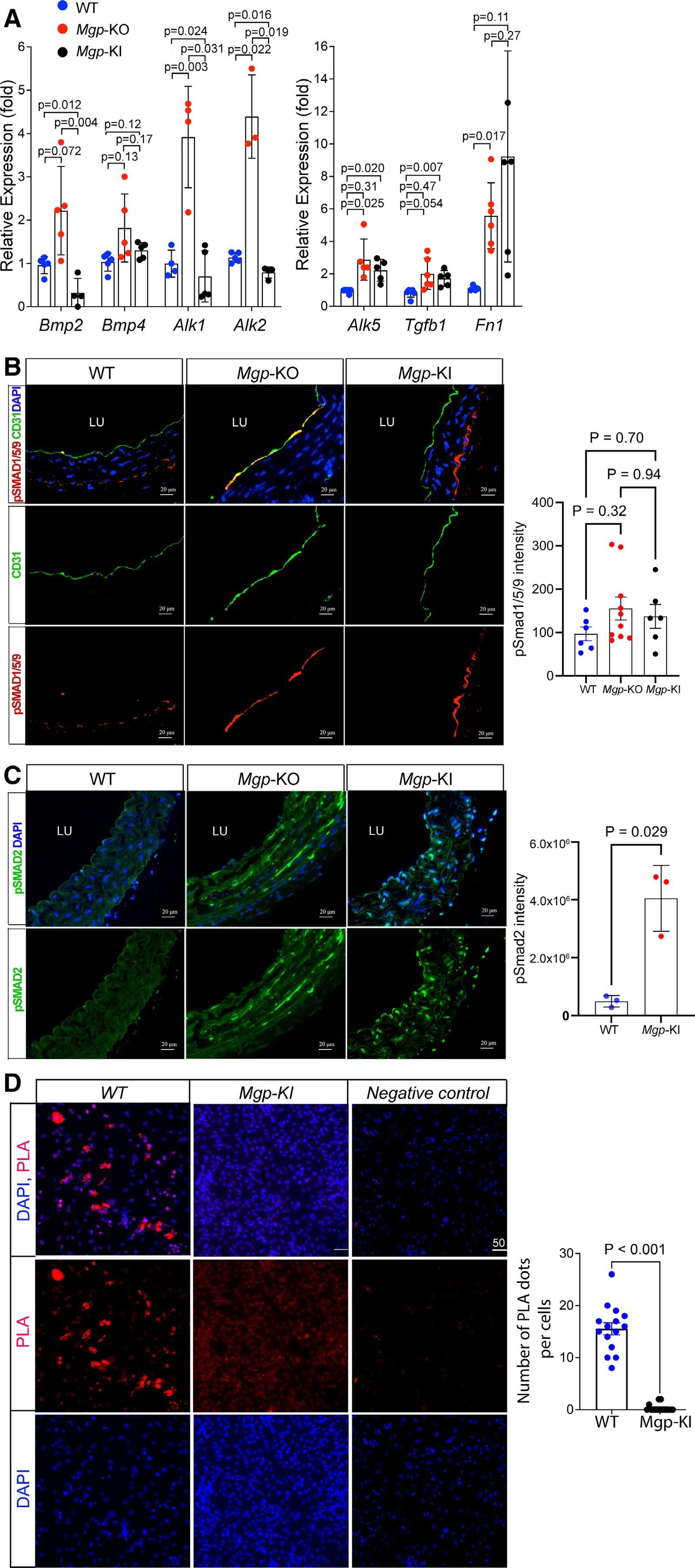
**BMP (bone morphogenetic protein) and TGFβ (transforming growth factor β) activation in the *Mgp* (matrix Gla protein)–knockin (KI) aorta. A**, Aortic expression of BMP components (*Bmp2*, *Bmp4*, *Alk1*, *Alk2*; **left**) and TGFβ components (*Alk5*, *Tgfb1*) and *Fn1* (**right**) in wild-type (WT), *Mgp*-knockout (KO), and *Mgp*-KI aortas at 4 weeks of age (n=5 mice per group; 3 males, 2 females). One-way ANOVA with Tukey post hoc test. **B**, Immunofluorescence for pSMAD (phosphorylated SMAD) 1/5/9 (red) and CD31 (green, **middle**) in aortas from WT, *Mgp*-knockout (KO), and *Mgp*-KI mice. pSMAD1/5/9 and CD31 showed colocalization (yellow) in the *Mgp*-KO aorta, but not in WT or *Mgp*-KI aortas. **C**, Immunofluorescence for pSMAD2 (green, middle) in aortas from WT, *Mgp*-KO, and *Mgp*-KI mice. Mineralization increased background staining in the *Mgp*-KO aortas. DAPI (4',6-diamidino-2-phenylindole) was used to visualize nuclei. Bars, 20 µm. Intensity determined by ImageJ (n=3 sections from male mice; 6 or 3 fields of view per section. One-way ANOVA with Tukey post hoc test, and unpaired 2-tailed Student *t* tests. **D**, Representative images and quantification of proximity ligation assay (PLA) to detect interaction between endogenous MGP and BMP4 in WT and *Mgp*-KI aortic cells. Negative control refers to anti-BMP4 antibodies only. Blue, DAPI; red, PLA signal. Bars: 50 µm. The quantification is shown as the number of PLA dots per cell (3 male and 3 female mice per group were used for the cell preparations). Unpaired 2-tailed Student *t* tests. LU indicates lumen.

Because the Pro64Gly mutation in *Mgp* appeared to have less effect on the vascular phenotype than complete *Mgp* deletion, we examined the BMP and TGFβ signaling in the aortic wall. We first assessed induction of the BMP components *Bmp2*, *Bmp4*, *Alk1*, and *Alk2*, known to be induced in the *Mgp*-knockout aortas.^6^ BMP signaling molecule expression was significantly increased in the *Mgp*-knockout aortas, but not in the *Mgp*-knockin aortas, when compared with WT aortas, as determined by qPCR (Figure 3A). To assess the location of the BMP activation, we immunostained the aortas with antibodies to pSMAD (phosphorylated SMAD) 1/5/9. Unexpectedly, the location of pSMAD1/5/9 was different between the aortas. The pSMAD1/5/9 staining was found peripherally at the transition between the media and adventitia, in the WT and *Mgp*-knockin aortas (Figure 3B). In contrast, the staining was clearly associated with the luminal endothelium in the *Mgp*-knockout aortas (Figure 3B), where it costained with the endothelial marker CD31. Although SMAD1/5/9 intensity appeared elevated in *Mgp*-knockin and *Mgp*-knockout aortas, the difference was not statistically significant. Thus, loss of the entire MGP protein moves the BMP activation to the endothelium, whereas BMP activation in the WT and *Mgp*-knockin is more restricted to the aortic periphery.

Based on the increased fibrosis detected by Masson Trichrome staining in the *Mgp*-knockin aortas, we also examined the expression of the TGFβ components *Alk5 and Tgfb1*. Previous studies have shown that in the absence of MGP, BMPs induce local expression of *Alk1*, which in turn enhances *Alk5* and HOXD3 expression, thereby enabling BMP and MGP to regulate TGFβ signaling.^38,39^ The results showed that *Tgfb1* and *Alk5* expression increased in the *Mgp*-knockin aortas together with that of *Fn1* (Figure 3A), which has been associated with fibrosis. To assess TGFβ signaling in the vascular media, we immunostained the aortas with antibodies to pSMAD2/3 and found it to be enhanced throughout the media and endothelium of the *Mgp*-knockin aortas compared with WT (Figure 3C). The staining for pSMAD2/3 was affected by the extensive mineralization in the *Mgp*-knockout aortas, which made it difficult to assess, but a mild increase was consistently observed. At the systemic level, serum TGFβ1 concentrations did not differ significantly between WT and *Mgp*-knockin mice (51.3±13.0 versus 42.2±12.1 ng/mL, *P*>0.05; Figure S2E). In contrast, serum BMP4 levels were significantly higher in *Mgp*-knockin mice compared with WT mice (337.9±24.2 versus 219.9±35.9 pg/mL, *P*=0.022; Figure S2F). These findings suggested that TGFβ signaling may be locally increased in *Mgp*-knockin aortas, whereas circulating BMP4 was systemically elevated in *Mgp*-knockin mice.

To determine whether a direct association between endogenous BMP4 and MGP occurs in vivo, we performed a proximity ligation assay using primary aortic cells isolated from both WT and *MGP*-knockin mice. Although there was a strong signal of interaction between BMP4 and MGP in WT aortic cells (Figure 3D), the signal was barely detectable in *MGP*-knockin aortic cells, consistent with the requirement for Pro64 to preserve the binding of MGP to BMP4.^10^

To define the mechanistic link between enhanced BMP signaling and fibrosis observed in the *Mgp*-knockin aorta, primary aortic cells from WT and *Mgp*-knockin mice were treated for 48 hours with BMP4 in the presence or absence of the TGFβ inhibitor SB431542 or recombinant TGFβ1 (Figure S3A through S3D). *Mgp*-knockin cells exhibited elevated *Bmp4* and *Mgp* expression compared with WT controls, which was attenuated by TGFβ inhibition (Figure S3B). Notably, exogenous TGFβ1 did not further augment *BMP4* or *Mgp* expression in *Mgp*-knockin cells, potentially representing a saturated effect in the setting of constitutively enhanced signaling. Consistent with activation of the TGFβ pathway, *Tgfβ2*, *Tgfβ3*, and *Alk5* were upregulated in the *Mgp*-knockin cells, with more pronounced increases in *Tgfβ2* and *Tgfβ3* (Figure S3C). SB431542 preferentially suppressed *Tgfβ2* and *Tgfβ3* expression to a greater extent compared with *Tgfβ1* and *Alk5*, possibly due to different sensitivity of these isoforms to receptor blockade. Expression of profibrotic genes, including *Col1a1*, *Col3a1*, *Col6a1*, and *Fn1*, was consistently elevated in the *Mgp*-knockin cells (Figure S3D). Although responses to TGFβ1 or SB431542 varied among targets, *Col1a1* expression was robustly reduced by TGF-β inhibition, which supports a functional contribution of TGFβ signaling to the fibrotic phenotype downstream of BMP activity.

Ki67 is widely used as a common marker for cell proliferation,^40^ and cleaved caspase 3 is used as an indicator for apoptotic signaling.^41^ The expression of both Ki67 and cleaved caspase 3 was increased in *Mgp*-knockout and *Mgp*-knockin aortas (Figure S4). Ki67 and cleaved caspase 3 expression was more limited to the adventitial side in *Mgp*-knockin aortas, whereas more staining was observed in both adventitial and medial layers in the *Mgp*-knockout aortas. Similar increases in Ki67 and cleaved caspase 3 expression were observed in a mouse model of aortic aneurysm,^42^ suggesting that dynamic cell turnover may occur during aortic remodeling.

### Transcriptional Profiles and Trajectory Analysis of Medial Cells From *Mgp*-Knockin Aortas

Our results pointed to potential changes in differentiation of the medial cells in a direction driven by abnormal BMP/TGFβ signaling. To further characterize the cellular and molecular changes induced in the *Mgp*-knockin aortas, we performed scRNA-seq analysis on whole aortas isolated from 4-week-old WT and *Mgp*-knockin mice of mixed sex.

**Figure 4.**
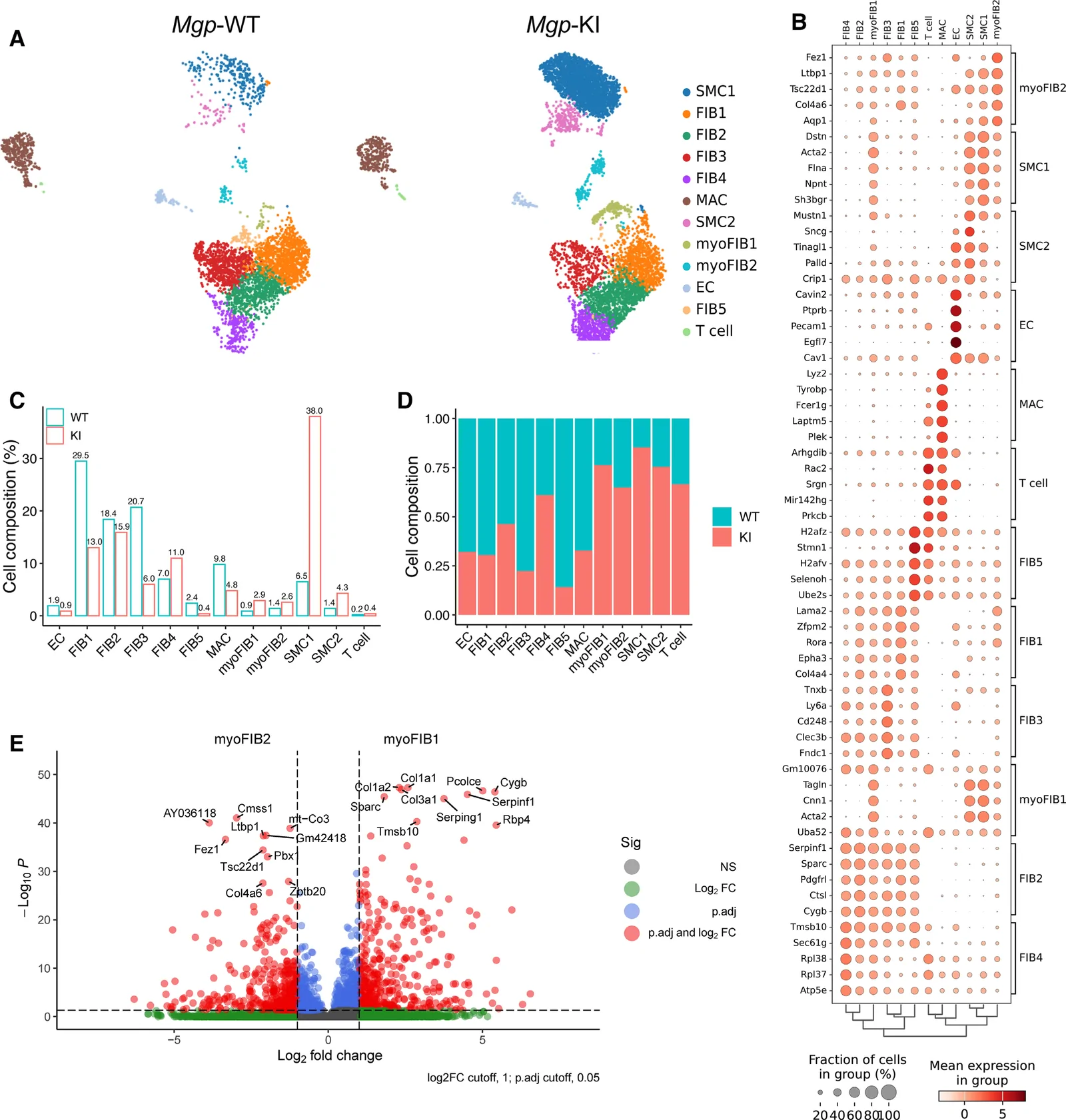
**Characterization of vascular cell differentiation between wild-type (WT) and *Mgp*-knockin (KI) aortas revealed by single-cell RNA-sequencing. A**, Uniform manifold approximation and projection (UMAP) plots displaying the major aortic cell populations colored by different cell types in WT and KI aortas. **B**, Dot plot illustrating the expression levels of top marker genes for each cell type identified in the UMAP plots. Dot size represents the fraction of cells within each group expressing the marker gene, and dot color intensity indicates the mean expression level. **C**, Column chart showing comparison of percentages of different cell types in WT and *Mgp*-KI aortas. **D**, Stacked bar plot showing the proportion of cells for each cell type between WT and *Mgp*-KI aortas. **E**, Volcano plot displaying differentially expressed genes in myofibroblast (myoFIB) 1 and myoFIB2 cell clusters. The *y* axis represents −log10 *P* values, whereas the *x* axis displays log2-fold change values. Eight mice (4 males, 4 females) per group were used for the cell preparations. EC indicates endothelial cells; FC, fold change; FIB, fibroblasts; MAC, macrophages; NS, not significant; P adj, adjusted *P* value; and SMC, smooth muscle cells.

The uniform manifold approximation and projection was used to visualize the single-cell expression data. Cluster-specific marker genes identified 12 different cell populations, including ECs (1 cluster), SMCs (2 clusters), fibroblasts (5 clusters), myofibroblasts (2 clusters), and immune cells (2 clusters; Figure 4A). A dot plot map of the top gene markers for each cell cluster is presented in Figure 4B. The expression of MGP and the difference in gene marker expression between WT and Mgp-knockin aorta are shown in Figure S5. When normalizing the number of cells in each cell type to the total cell count, we observed a significant increase in the fraction of SMCs in the *Mgp*-knockin aorta compared with WT aorta (42.3% versus 7.9%), accompanied by a significant reduction in the fibroblast fraction (46.3% in *Mgp*-knockin versus 78% in WT; Figure 4C). In addition, we found that the *Mgp*-knockin aorta contained more myofibroblasts compared with the WT aorta (5.5% versus 2.3%). The stacked bar graph displays the relative proportions of each cell type in the WT and *Mgp*-knockin aorta (Figure 4D), which revealed increased proportions of SMCs and myofibroblasts but a decreased proportion of fibroblasts in the *Mgp*-knockin aorta. The myofibroblast populations, located between the SMC and fibroblast populations in the uniform manifold approximation and projection, exhibited representative marker genes for both SMCs and fibroblasts with differential expression levels (Figure 4B and 4E; Figure S4C). BMP and TGFβ signaling molecules showed distinctive expression patterns in aortic cell populations (Figure S6). ECs displayed enriched BMP4, BMP6, and TGFβ1 expression compared with other cell clusters. In addition, FIB5 contained relatively high expression levels of BMP4 and TGFβ1.

**Figure 5.**
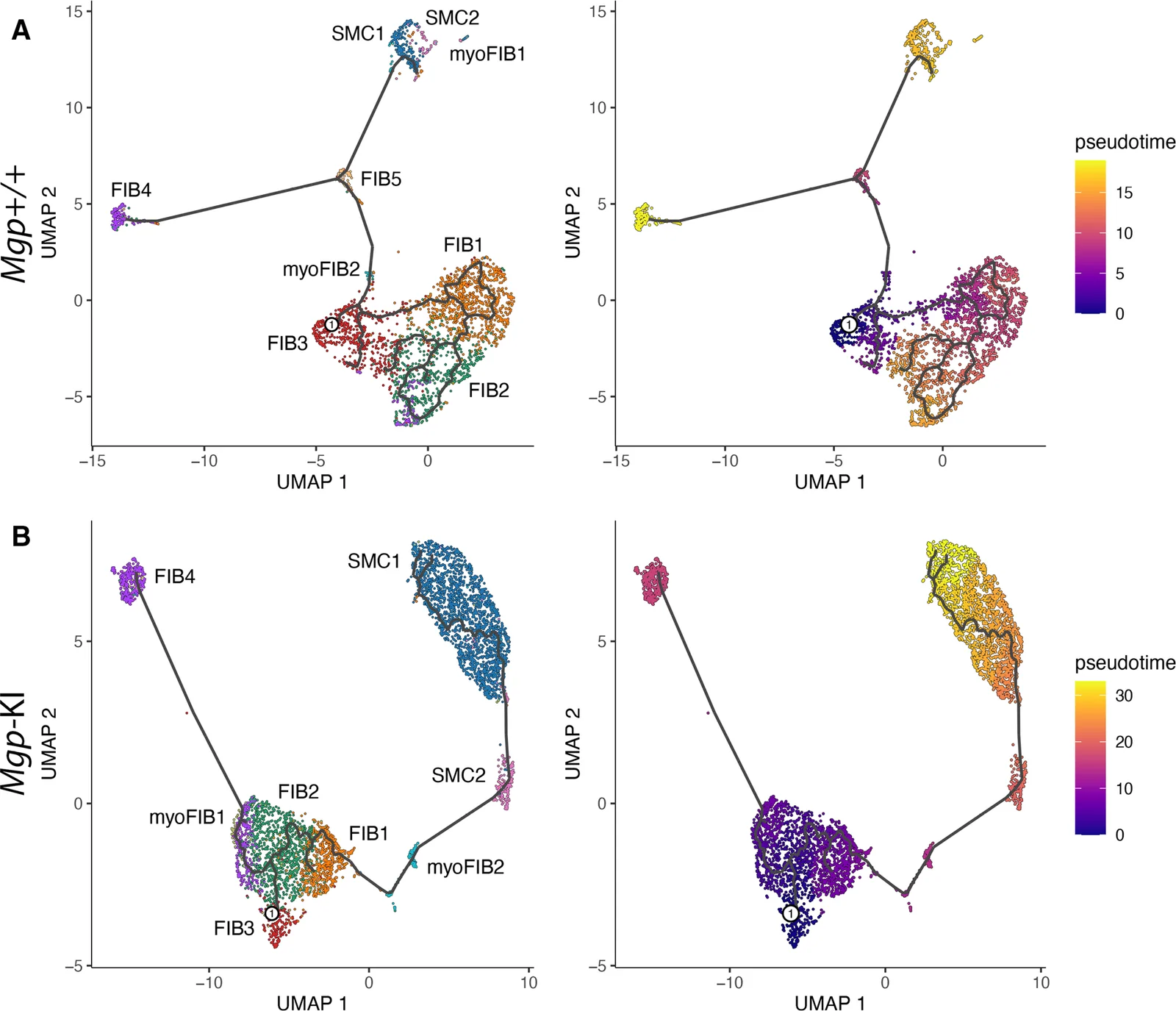
**Single-cell trajectory reconstruction and pseudotime analysis.** Single-cell trajectory and pseudotime analyses of the cell clusters in (A) wild-type and (B) Mgp-knockin (KI) aortas, reconstructed by Monocle3. **Left**, Cells are colored by clusters. **Right**, Cells are colored by pseudotime. FIB indicates fibroblasts; myoFIB, myofibroblasts; SMC, smooth muscle cells; and UMAP, uniform manifold approximation and projection.

We then performed pseudotime-assisted trajectory analyses using the R Package Monocle to understand the percentage changes and cell transitions in cell populations. We excluded immune cells and ECs in the analyses. The trajectory analyses demonstrated that in the WT aorta, myofibroblast cluster 2 transitioned to fibroblast cluster 5, which represented an intermediate transition phenotype that led to SMC clusters (Figure 5A). In contrast, fibroblast cluster 5 was diminished in the *Mgp*-knockin aorta, and myofibroblast cluster 2 acted as the direct transitioning cell cluster between fibroblast clusters and SMC clusters (Figure 5B). The results suggest that the direct transition from fibroblasts to SMCs via myofibroblast cluster 2 promotes SMC differentiation and proliferation, which would explain the increased fraction of SMCs in the *Mgp*-knockin aortas.

We next examined SMC, myofibroblast, and fibroblast/mesenchymal progenitor lineage markers in primary aortic cells from WT and *Mgp*-knockin mice (Figure S7A through S7C). *Pdgfrα*, *Acta2*, *Tagln*, and *Myh11* were significantly upregulated in *Mgp*-knockin cells, and BMP4 treatment did not further enhance their expression. The TGFβ inhibitor SB431542 alleviated the increased expression of *Acta2*, *Tagln*, and *Myh11* in *Mgp*-knockin cells, but did not affect elevated *Pdgfrα* levels. Similarly, exogenous TGFβ1 modestly suppressed *Acta2*, *Tagln*, and *Myh11* expression without altering *Pdgfrα*. These findings suggest that SMC/myofibroblast gene upregulation in *Mgp*-knockin cells is TGFβ-dependent, whereas fibroblast/mesenchymal progenitor *Pdgfrα* expression is likely regulated through a distinct mechanism.

### High Phosphate Feeding Did Not Trigger Vascular Calcification in *Mgp*-Knockin Mice

To examine whether a high-phosphate diet promoted vascular calcification in the *Mgp*-knockin mice similar to previous reports on mice with other mutant forms of MGP,^12^ we fed the *Mgp*-knockin and WT controls a high-phosphate (2%) diet for 12 weeks. The results showed no mineralization in any of the aortas of the phosphate-fed mice, as determined by microcomputed tomography and histochemical staining (Figure S8). Thus, even with high phosphate feeding, vascular calcification was not triggered in the Mgp-knockin mice, consistent with previous reports that the phosphorylated serine residues in the N-terminus of MGP, which are present in the *Mgp*-knockin mice, protect against mineralization.^12^

### Development of AVMs in *Mgp*-Knockin Mice

Dysregulation of BMP activity is well known to drive the formation of AVMs, which are characteristic of HHT and caused by mutations in the Bmp9/10, *Alk1*, *Eng*, or *Smad4* genes.^43^ AVMs are also a consistent feature in the *Mgp*-knockout mice, which develop AVMs in several locations, such as the lungs, kidney, retina, brain, and brown adipose tissue.^15,17,18^ The development of AVMs is strongly associated with enhanced endothelial BMP activity.^44^ Furthermore, excess MGP from a transgene counteracts the AVMs in *Alk1*^+/^^−^ mice, a model of hereditary hemorrhagic telangiectasia 2.^13^

**Figure 6.**
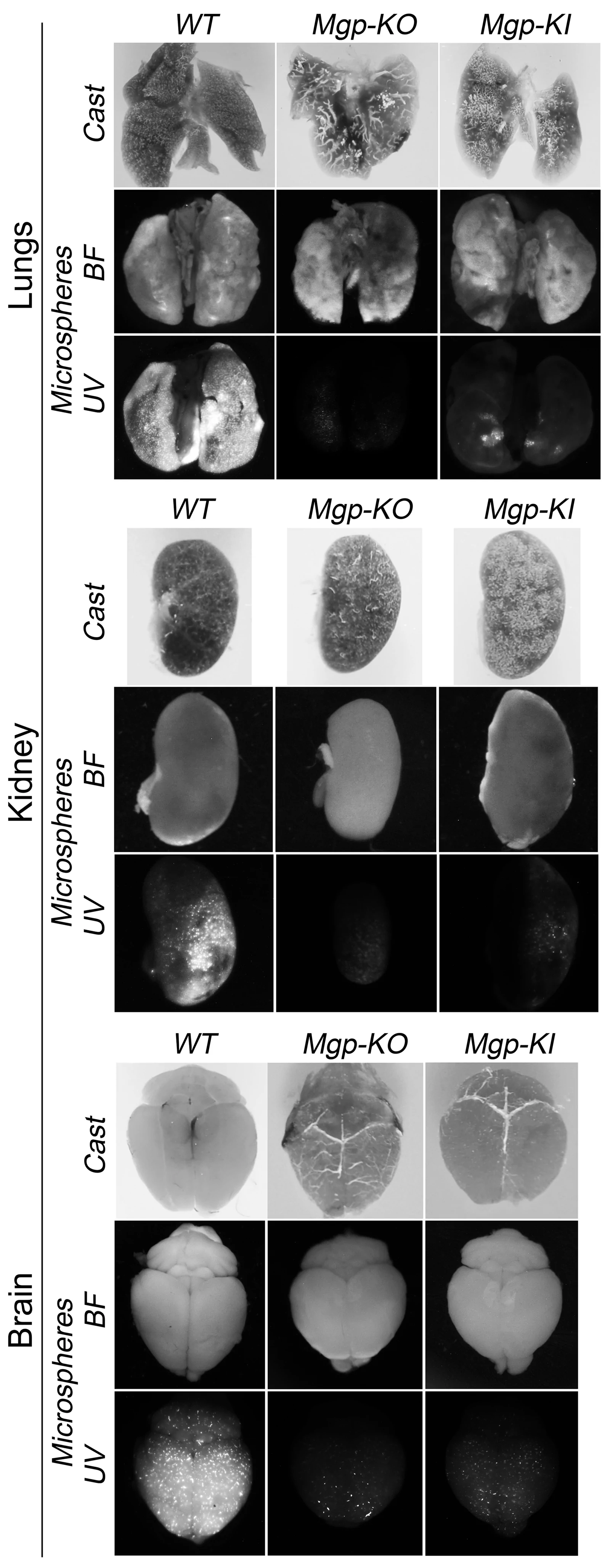
**Arteriovenous malformations (AVMs) in the *Mgp*-knockin (KI) mouse. Top**, For each organ: vascular casts from lungs, kidneys, and brain in wild-type (WT), *Mgp*-knockout (KO), and *Mgp*-KI mice. **Middle** and **bottom**, For each organ: imaging performed after microsphere perfusion in lungs, kidneys, and brain in WT, *Mgp*-KO, and *Mgp*-KI mice. Lack of visualization under ultraviolet (UV) light indicates the presence of AVMs in the vasculature (n=3 mice were analyzed per group; 1 male, 2 females). BF indicates bright-field.

To investigate whether AVMs also develop in the *Mgp*-knockin mice, we performed casting of the vasculature or perfusion of GFP-fluorescent beads before examining lungs, kidneys, and brain. All 3 organs showed abnormal vascular casts and perfusion of fluorescent beads (Figure 6) in all mice examined. *Mgp*-knockout mice were included for comparison. We confirmed the presence of enlarged vessels in the Mgp-knockin by immunofluorescence for CD31 (Figure S9). We also assessed the expression of EndMT markers in lungs, kidneys, and brain by qPCR. The results showed expression of EndMT markers in the various organs, but with some variation. *NCdh*, *Sox2*, *Cd90*, and *Oct4* were mostly expressed in lungs and kidneys (Figure S10), whereas *Cd44* was prominent in the brain. It suggests that EndMTs contribute to the AVMs in the *Mgp*-knockin mice and that the elasto-protection afforded by the mutated MGP in the aorta does not protect against AVMs. No induction of *Runx2* was detected.

## Discussion

In this study, we examined the in vivo significance of abolishing BMP binding in MGP by mutating Pro64. We found that without this regulatory function, BMP activity was enhanced on the adventitial side of the media. Furthermore, the mutation disrupted the balance between BMP and TGFβ signaling and promoted TGFβ activity, increasing the fraction of myofibroblasts resulting in fibrosis. The study adds novel information on MGP function, separating the BMP regulation from previous findings of elasto-protective actions by the N-terminal serine residues in MGP.^12,45^ These serines have not been implicated in BMP binding,^10^ and were not mutated in our study. The modulation of BMP activity was also found to be essential in preventing AVMs.

The MD studies supported that introduction of the Pro64Gly mutation suppressed the conformational changes in MGP required to bind BMP4, whereas the ability to bind Ca^2+^ remained unchanged, consistent with previous in vitro studies.^10^ Furthermore, the structural landscapes for Ca^2+^ binding of MGP, both with Pro64Gly and Pro64Phe mutations, resemble the crystallized structural features of osteocalcin.^46^ Therefore, changes in calcium binding are unlikely to be responsible for the changes in cell differentiation observed in the *Mgp*-knockin aorta. Mechanistically, proximity ligation assay assays demonstrated that MGP interacts directly with BMP4, an interaction that is significantly reduced with the Pro64Gly-MGP mutant protein. Loss of effective MGP-BMP4 binding in the mutant likely impairs the normal negative feedback regulation of BMP signaling,^18^ resulting in sustained pathway activation. Consistent with this model, expression of both *Bmp4* and the mutated *Mgp* was upregulated, supporting a feed-forward amplification of BMP signaling in *Mgp*-knockin cells. In addition, this upregulation was effectively blocked by TGFβ inhibition, even in the presence of exogenous BMP4, suggesting TGFβ signaling acts downstream or in concert with BMP activity to reinforce and sustain the signaling loop. In primary aortic cells, enhanced BMP signaling in *Mgp*-knockin cells promotes profibrotic gene expression, likely through the activation of the TGFβ pathway to sustain the fibrotic phenotype.

Our results showed increased BMP activity on the adventitial side of the *Mgp*-knockin media, similar in location to wild type, but opposite to the endothelial BMP activation in the *Mgp*-knockout aorta. Interestingly, the adventitia also showed evidence of enhanced proliferation and apoptosis in *Mgp*-knockout and *Mgp*-knockin aortas. Such dynamic changes in cell populations have similarly been reported during aortic remodeling in 2 models of aortic aneurysm,^41,42^ suggesting that increased cellular turnover may contribute to active vascular remodeling. BMPs are known to play important roles in adventitia,^47^ potentially supporting maintenance of the aortic wall. BMP4 has an important role in initiating SMC differentiation^48,49^ and has been used as an obligatory time-limited step in protocols for induction of SMC lineage in stem cells.^21^ BMP4 also induces *Alk1* in vascular cells,^50,51^ which in turn can trigger the expression of *Alk5*,^15,38^ with subsequent TGFβ signaling and SMC marker induction.^38,50^ Therefore, the absence of functional MGP would be expected to lead to enhanced *Alk5* expression.^15^ Activated ALK1 has also been shown to induce *Tgfb1* expression through HOXD3 and BMP9 signaling,^39^ which would promote TGFβ signaling even further. The altered mural cell composition favoring fibrosis is consistent with the promotion of TGFβ signaling.

The internal elastic lamina and the medial elastic lamellae appeared intact in the *Mgp*-knockin aorta, and calcification was not induced even with a high phosphate diet. Protection of elastin by MGP has been described by Parashar et al^12,45^ to reduce calcification by maintaining the N-terminal phosphorylated serine residues in MGP. The authors were also successful in limiting mineralization in the *Mgp*-knockout mice by reducing the aortic elastin level.^52^ These N-terminal serines were left intact in the *Mgp*-knockin mice, which would explain the lack of calcification. However, the medial cell differentiation was not analyzed further in these reports, which limits the comparison to our study.

Diabetes is known to be associated with degradation of elastic lamellae,^53^ which might play a role in the increased propensity for calcification in diabetic subjects. Indeed, Cheng et al^54^ demonstrated that diabetic-induced BMP activity enhanced adventitial myofibroblast activation, osteogenesis and calcification through a BMP2-Msx2 axis in a mouse model of hyperlipidemia and diabetes. In addition, Simionescu et al^55^ showed that EDPs trigger myofibroblasts to undergo osteogenesis and calcification. MGP was not studied in these scenarios, but would be expected to counteract both altered differentiation and elastin degradation.

AVMs were detected in several organs in the *Mgp*-knockin mouse, including the lungs, kidneys, and brain. This supports that BMP regulation is a major factor in preventing AVMs, because mice with global or endothelial-specific *Mgp* gene deletion develop AVMs^15,44^ similar to heterozygous *Alk1* deficiency.^13^ Mgp is also expressed in pericytes,^56^ which are important analogues to SMCs in the microvasculature that may be involved in AVMs.^57^ However, additional studies are needed to clarify the role of MGP derived from pericytes in microvascular networks.

Strengths of our study include the successful separation of BMP regulation from the protection of elastin, allowing us to identify unique BMP effects in vivo. We were able to apply AlphaFold3 and MD simulations to MGP, which were consistent with previous and current results in cells and mice. Modulation of cell composition may be a parameter that could be applied to improve understanding of other types of aortic disorders.

Limitations include the use of only 4-week-old mice, mainly due to the short lifespans of the *Mgp*-knockout mice, which did not allow us to monitor the progress of cell differentiation over time. It was further complicated by the lack of unique marker genes for individual cell clusters, preventing lineage tracing. BMP actions are also difficult to follow because they are highly specific to time and location. Furthermore, we did not determine sex differences and interactions because we were unable to detect differences between male and female mice or cells, possibly due to the use of 4-week old mice. Changing the focus to older mice and expanding the study may be required to fully investigate potential sex differences.

We propose a working model for MGP that has at least 4 functional aspects: (1) protection of elastin, (2) binding and regulation of BMP, (3) downstream regulation of TGFβ activation, and (4) interaction with the extracellular matrix. Global knockout of MGP activates BMP signaling on the endothelial side, triggering transitions in ECs and SMCs, which contribute to calcifying cells. EndMTs involve proteolysis of the elastic lamellae, which generate EDPs that promote calcification in fibroblast-like cells. Selective loss of the BMP inhibition in MGP enhances BMP signaling at the media-adventitia transition, in turn increasing the TGFβ signaling and myofibroblast fraction in the cell composition. Normal MGP may also have a morphogenic function that supports the layered architecture of the aorta by interacting with the extracellular matrix to anchor cells, while differentiation is being regulated through BMP and TGFβ signaling. In conclusion, our new data highlight that MGP relies on separate but complementary functions, such as BMP binding and elastin protection, to ensure the integrity of the vasculature.

## ARTICLE INFORMATION

### Disclosures

None.

### Supplemental Material

Figures S1–S10

Major Resources Table

ARRIVE Guidelines

## Supplementary Material

## References

[1] Price PA Fraser JD Metz-Virca G. Molecular cloning of matrix Gla protein: implications for substrate recognition by the vitamin K-dependent gamma-carboxylase. Proc Natl Acad Sci U S A. 1987;84:8335–8339. doi: 10.1073/pnas.84.23.8335 3317405 10.1073/pnas.84.23.8335 PMC299537

[2] Hauschka PV Lian JB Cole DE Gundberg CM. Osteocalcin and matrix Gla protein: vitamin K-dependent proteins in bone. Physiol Rev. 1989;69:990–1047. doi: 10.1152/physrev.1989.69.3.990 2664828 10.1152/physrev.1989.69.3.990

[3] Luo G Ducy P McKee MD Pinero GJ Loyer E Behringer RR Karsenty G. Spontaneous calcification of arteries and cartilage in mice lacking matrix GLA protein. Nature. 1997;386:78–81. doi: 10.1038/386078a0 9052783 10.1038/386078a0

[4] Leroux-Berger M Queguiner I Maciel TT Ho A Relaix F Kempf H. Pathological calcification of adult vascular smooth muscle cells differs upon their crest or mesodermal embryonic origin. J Bone Miner Res. 2011;26:1543. doi: 10.1002/jbmr.382 21425330 10.1002/jbmr.382

[5] Yao Y Jumabay M Ly A Radparvar M Cubberly MR Bostrom KI. A role for the endothelium in vascular calcification. Circ Res. 2013;113:495–504. doi: 10.1161/CIRCRESAHA.113.301792 23852538 10.1161/CIRCRESAHA.113.301792 PMC3851028

[6] Yao J Guihard PJ Blazquez-Medela AM Guo Y Moon JH Jumabay M Bostrom KI Yao Y. Serine protease activation essential for endothelial-mesenchymal transition in vascular calcification. Circ Res. 2015;117:758–769. doi: 10.1161/CIRCRESAHA.115.306751 26265629 10.1161/CIRCRESAHA.115.306751 PMC4600461

[7] Speer MY Yang HY Brabb T Leaf E Look A Lin WL Frutkin A Dichek D Giachelli CM. Smooth muscle cells give rise to osteochondrogenic precursors and chondrocytes in calcifying arteries. Circ Res. 2009;104:733–741. doi: 10.1161/CIRCRESAHA.108.183053 19197075 10.1161/CIRCRESAHA.108.183053 PMC2716055

[8] Beazley KE Reckard S Nurminsky D Lima F Nurminskaya M. Two sides of MGP null arterial disease: chondrogenic lesions dependent on transglutaminase 2 and elastin fragmentation associated with induction of adipsin. J Biol Chem. 2013;288:31400–31408. doi: 10.1074/jbc.M113.495556 24036114 10.1074/jbc.M113.495556 PMC3829453

[9] Schurgers LJ Spronk HM Skepper JN Hackeng TM Shanahan CM Vermeer C Weissberg PL Proudfoot D. Post-translational modifications regulate matrix Gla protein function: importance for inhibition of vascular smooth muscle cell calcification. J Thromb Haemost. 2007;5:2503–2511. doi: 10.1111/j.1538-7836.2007.02758.x 17848178 10.1111/j.1538-7836.2007.02758.x

[10] Yao Y Shahbazian A Bostrom KI. Proline and gamma-carboxylated glutamate residues in matrix Gla protein are critical for binding of bone morphogenetic protein-4. Circ Res. 2008;102:1065–1074. doi: 10.1161/CIRCRESAHA.107.166124 18369157 10.1161/CIRCRESAHA.107.166124

[11] Wei FF Trenson S Verhamme P Vermeer C Staessen JA. Vitamin K-dependent matrix Gla protein as multifaceted protector of vascular and tissue integrity. Hypertension. 2019;73:1160–1169. doi: 10.1161/HYPERTENSIONAHA.119.12412 31006332 10.1161/HYPERTENSIONAHA.119.12412 PMC6510326

[12] Parashar A Bak K Murshed M. Prevention of arterial elastocalcinosis: differential roles of the conserved glutamic acid and serine residues of matrix Gla protein. Arterioscler Thromb Vasc Biol. 2022;42:e155–e167. doi: 10.1161/ATVBAHA.122.317518 35418245 10.1161/ATVBAHA.122.317518

[13] Bostrom KI Guihard P Blazquez Medela AM Yao J Moon JH Penton A Yao Y. Matrix Gla protein limits pulmonary arteriovenous malformations in ALK1 deficiency. Eur Respir J. 2015;45:849–852. doi: 10.1183/09031936.00114714 25614167 10.1183/09031936.00114714 PMC4373345

[14] Yao J Guihard PJ Wu X Blazquez-Medela AM Spencer MJ Jumabay M Tontonoz P Fogelman AM Bostrom KI Yao Y. Vascular endothelium plays a key role in directing pulmonary epithelial cell differentiation. J Cell Biol. 2017;216:3369–3385. doi: 10.1083/jcb.201612122 28838957 10.1083/jcb.201612122 PMC5626536

[15] Yao Y Jumabay M Wang A Bostrom KI. Matrix Gla protein deficiency causes arteriovenous malformations in mice. J Clin Invest. 2011;121:2993–3004. doi: 10.1172/JCI57567 21765215 10.1172/JCI57567 PMC3148746

[16] Yao Y Nowak S Yochelis A Garfinkel A Bostrom KI. Matrix GLA protein, an inhibitory morphogen in pulmonary vascular development. J Biol Chem. 2007;282:30131–30142. doi: 10.1074/jbc.M704297200 17670744 10.1074/jbc.M704297200

[17] Yao Y Yao J Radparvar M Blazquez-Medela AM Guihard PJ Jumabay M Bostrom KI. Reducing Jagged 1 and 2 levels prevents cerebral arteriovenous malformations in matrix Gla protein deficiency. Proc Natl Acad Sci USA. 2013;110:19071–19076. doi: 10.1073/pnas.1310905110 24191040 10.1073/pnas.1310905110 PMC3839731

[18] Guihard PJ Guo Y Wu X Zhang L Yao J Jumabay M Yao Y Garfinkel A Bostrom KI. Shaping waves of bone morphogenetic protein inhibition during vascular growth. Circ Res. 2020;127:1288–1305. doi: 10.1161/CIRCRESAHA.120.317439 32854559 10.1161/CIRCRESAHA.120.317439 PMC7987130

[19] Kulikauskas MR X S Bautch VL. The versatility and paradox of BMP signaling in endothelial cell behaviors and blood vessel function. Cell Mol Life Sci. 2022;79:77. doi: 10.1007/s00018-021-04033-z 35044529 10.1007/s00018-021-04033-z PMC8770421

[20] Watterston C Zeng L Onabadejo A Childs SJ. MicroRNA26 attenuates vascular smooth muscle maturation via endothelial BMP signalling. PLoS Genet. 2019;15:e1008163. doi: 10.1371/journal.pgen.1008163 31091229 10.1371/journal.pgen.1008163 PMC6538191

[21] Shen M Quertermous T Fischbein MP Wu JC. Generation of vascular smooth muscle cells from induced pluripotent stem cells: methods, applications, and considerations. Circ Res. 2021;128:670–686. doi: 10.1161/CIRCRESAHA.120.318049 33818124 10.1161/CIRCRESAHA.120.318049 PMC10817206

[22] Mailleux AA Kelly R Veltmaat JM De Langhe SP Zaffran S Thiery JP Bellusci S. Fgf10 expression identifies parabronchial smooth muscle cell progenitors and is required for their entry into the smooth muscle cell lineage. Development. 2005;132:2157–2166. doi: 10.1242/dev.01795 15800000 10.1242/dev.01795

[23] Goumans MJ Ten Dijke P. TGF-β signaling in control of cardiovascular function. Cold Spring Harb Perspect Biol. 2018;10:a022210. doi: 10.1101/cshperspect.a022210 28348036 10.1101/cshperspect.a022210 PMC5793760

[24] Abramson J Adler J Dunger J Evans R Green T Pritzel A Ronneberger O Willmore L Ballard AJ Bambrick J. Accurate structure prediction of biomolecular interactions with AlphaFold 3. Nature. 2024;630:493–500. doi: 10.1038/s41586-024-07487-w 38718835 10.1038/s41586-024-07487-w PMC11168924

[25] Jo S Kim T Iyer VG Im W. CHARMM-GUI: a web-based graphical user interface for CHARMM. J Comput Chem. 2008;29:1859–1865. doi: 10.1002/jcc.20945 18351591 10.1002/jcc.20945

[26] Case DA Aktulga HM Belfon K Cerutti DS Cisneros GA Cruzeiro VWD Forouzesh N Giese TJ Götz AW Gohlke H. AmberTools. J Chem Inf Model. 2023;63:6183–6191. doi: 10.1021/acs.jcim.3c01153 37805934 10.1021/acs.jcim.3c01153 PMC10598796

[27] Roe DR Cheatham TE 3rd. PTRAJ and CPPTRAJ: software for processing and analysis of molecular dynamics trajectory data. J Chem Theory Comput. 2013;9:3084–3095. doi: 10.1021/ct400341p 26583988 10.1021/ct400341p

[28] Scherer MK Trendelkamp-Schroer B Paul F Perez-Hernandez G Hoffmann M Plattner N Wehmeyer C Prinz JH Noe F. PyEMMA 2: a software package for estimation, validation, and analysis of Markov models. J Chem Theory Comput. 2015;11:5525–5542. doi: 10.1021/acs.jctc.5b00743 26574340 10.1021/acs.jctc.5b00743

[29] Yao J Ma F Zhang L Zhu C Jumabay M Yao Z Wang L Cai X Zhang D Qiao X. Single-cell RNA-Seq identifies dynamic cardiac transition program from ADCs induced by leukemia inhibitory factor. Stem Cells. 2022;40:932–948. doi: 10.1093/stmcls/sxac048 35896368 10.1093/stmcls/sxac048 PMC9585902

[30] Debaize L Jakobczyk H Rio AG Gandemer V Troadec MB. Optimization of proximity ligation assay (PLA) for detection of protein interactions and fusion proteins in non-adherent cells: application to pre-B lymphocytes. Mol Cytogenet. 2017;10:27. doi: 10.1186/s13039-017-0328-2 28736577 10.1186/s13039-017-0328-2 PMC5520345

[31] Zhao Y Yang Y Wu X Zhang L Cai X Ji J Chen S Vera A Bostrom KI Yao Y. CDK1 inhibition reduces osteogenesis in endothelial cells in vascular calcification. JCI Insight. 2024;9:e176065. doi: 10.1172/jci.insight.176065 38456502 10.1172/jci.insight.176065 PMC10972591

[32] Stout DB Chatziioannou AF Lawson TP Silverman RW Gambhir SS Phelps ME. Small animal imaging center design: the facility at the UCLA Crump Institute for Molecular Imaging. Mol Imaging Biol. 2005;7:393–402. doi: 10.1007/s11307-005-0015-2 16261425 10.1007/s11307-005-0015-2 PMC3005624

[33] Salvador J Hernandez GE Ma F Abrahamson CW Pellegrini M Goldman R Ridge KM Iruela-Arispe ML. Transcriptional evaluation of the ductus arteriosus at the single-cell level uncovers a requirement for vim (vimentin) for complete closure. Arterioscler Thromb Vasc Biol. 2022;42:732–742. doi: 10.1161/ATVBAHA.121.317172 35443793 10.1161/ATVBAHA.121.317172 PMC9806842

[34] Hao Y Stuart T Kowalski MH Choudhary S Hoffman P Hartman A Srivastava A Molla G Madad S Fernandez-Granda C. Dictionary learning for integrative, multimodal and scalable single-cell analysis. Nat Biotechnol. 2024;42:293–304. doi: 10.1038/s41587-023-01767-y 37231261 10.1038/s41587-023-01767-y PMC10928517

[35] Qiu X Mao Q Tang Y Wang L Chawla R Pliner HA Trapnell C. Reversed graph embedding resolves complex single-cell trajectories. Nat Methods. 2017;14:979–982. doi: 10.1038/nmeth.4402 28825705 10.1038/nmeth.4402 PMC5764547

[36] Hackeng TM Rosing J Spronk HM Vermeer C. Total chemical synthesis of human matrix Gla protein. Protein Sci. 2001;10:864–870. doi: 10.1110/ps.44701 11274477 10.1110/ps.44701 PMC2373974

[37] Hoang QQ Sicheri F Howard AJ Yang DS. Bone recognition mechanism of porcine osteocalcin from crystal structure. Nature. 2003;425:977–980. doi: 10.1038/nature02079 14586470 10.1038/nature02079

[38] Shao ES Lin L Yao Y Bostrom KI. Expression of vascular endothelial growth factor is coordinately regulated by the activin-like kinase receptors 1 and 5 in endothelial cells. Blood. 2009;114:2197–2206. doi: 10.1182/blood-2009-01-199166 19506300 10.1182/blood-2009-01-199166 PMC2744576

[39] Wang L Yao J Yu T Zhang D Qiao X Yao Z Wu X Zhang L Bostrom KI Yao Y. Homeobox D3, a novel link between bone morphogenetic protein 9 and transforming growth factor beta 1 signaling. J Mol Biol. 2020;432:2030–2041. doi: 10.1016/j.jmb.2020.01.043 32061928 10.1016/j.jmb.2020.01.043 PMC7225048

[40] Whitfield ML George LK Grant GD Perou CM. Common markers of proliferation. Nat Rev Cancer. 2006;6:99–106. doi: 10.1038/nrc1802 16491069 10.1038/nrc1802

[41] Emrich FC Okamura H Dalal AR Penov K Merk DR Raaz U Hennigs JK Chin JT Miller MO Pedroza AJ. Enhanced caspase activity contributes to aortic wall remodeling and early aneurysm development in a murine model of Marfan syndrome. Arterioscler Thromb Vasc Biol. 2015;35:146–154. doi: 10.1161/ATVBAHA.114.304364 25359856 10.1161/ATVBAHA.114.304364

[42] Bhamidipati CM Mehta GS Lu G Moehle CW Barbery C DiMusto PD Laser A Kron IL Upchurch GR Jr Ailawadi G. Development of a novel murine model of aortic aneurysms using peri-adventitial elastase. Surgery. 2012;152:238–246. doi: 10.1016/j.surg.2012.02.010 22828146 10.1016/j.surg.2012.02.010 PMC3601193

[43] Al Tabosh T Al Tarrass M Tourvieilhe L Guilhem A Dupuis-Girod S Bailly S. Hereditary hemorrhagic telangiectasia: from signaling insights to therapeutic advances. J Clin Invest. 2024;134:e176379. doi: 10.1172/JCI176379 38357927 10.1172/JCI176379 PMC10866657

[44] Yang Y Wu X Zhao Y Zhang D Zhang L Cai X Ji J Jing Z Bostrom KI Yao Y. Arterial-lymphatic-like endothelial cells appear in hereditary hemorrhagic telangiectasia 2 and contribute to vascular leakage and arteriovenous malformations. Circulation. 2025;151:299–317. doi: 10.1161/CIRCULATIONAHA.124.070925 39429196 10.1161/CIRCULATIONAHA.124.070925 PMC11789604

[45] Parashar A Gourgas O Lau K Li J Muiznieks L Sharpe S Davis E Cerruti M Murshed M. Elastin calcification in in vitro models and its prevention by MGP’s N-terminal peptide. J Struct Biol. 2021;213:107637. doi: 10.1016/j.jsb.2020.107637 33059036 10.1016/j.jsb.2020.107637

[46] Ami D Santambrogio C Vertemara J Bovio F Santisteban-Veiga A Sabin J Zampella G Grandori R Cipolla L Natalello A. The landscape of osteocalcin proteoforms reveals distinct structural and functional roles of its carboxylation sites. J Am Chem Soc. 2024;146:27755–27769. doi: 10.1021/jacs.4c09732 39348444 10.1021/jacs.4c09732

[47] Dyer L Wu Y Moser M Patterson C. BMPER-induced BMP signaling promotes coronary artery remodeling. Dev Biol. 2014;386:385–394. doi: 10.1016/j.ydbio.2013.12.019 24373957 10.1016/j.ydbio.2013.12.019 PMC4112092

[48] Yang P Troncone L Augur ZM Kim SSJ McNeil ME Yu PB. The role of bone morphogenetic protein signaling in vascular calcification. Bone. 2020;141:115542. doi: 10.1016/j.bone.2020.115542 32736145 10.1016/j.bone.2020.115542 PMC8185454

[49] Nakaoka T Gonda K Ogita T Otawara-Hamamoto Y Okabe F Kira Y Harii K Miyazono K Takuwa Y Fujita T. Inhibition of rat vascular smooth muscle proliferation in vitro and in vivo by bone morphogenetic protein-2. J Clin Invest. 1997;100:2824–2832. doi: 10.1172/JCI119830 9389748 10.1172/JCI119830 PMC508488

[50] Yao Y Zebboudj AF Torres A Shao E Bostrom K. Activin-like kinase receptor 1 (ALK1) in atherosclerotic lesions and vascular mesenchymal cells. Cardiovasc Res. 2007;74:279–289. doi: 10.1016/j.cardiores.2006.09.014 17074310 10.1016/j.cardiores.2006.09.014

[51] Yao Y Zebboudj AF Shao E Perez M Bostrom K. Regulation of bone morphogenetic protein-4 by matrix GLA protein in vascular endothelial cells involves activin-like kinase receptor 1. J Biol Chem. 2006;281:33921–33930. doi: 10.1074/jbc.M604239200 16950789 10.1074/jbc.M604239200

[52] Khavandgar Z Roman H Li J Lee S Vali H Brinckmann J Davis EC Murshed M. Elastin haploinsufficiency impedes the progression of arterial calcification in MGP-deficient mice. J Bone Miner Res. 2014;29:327–337. doi: 10.1002/jbmr.2039 23857752 10.1002/jbmr.2039

[53] Guihard PJ Yao J Blazquez-Medela AM Iruela-Arispe L Bostrom KI Yao Y. Endothelial-mesenchymal transition in vascular calcification of Ins2Akita/+ mice. PLoS One. 2016;11:e0167936. doi: 10.1371/journal.pone.0167936 27936229 10.1371/journal.pone.0167936 PMC5148029

[54] Cheng SL Shao JS Charlton-Kachigian N Loewy AP Towler DA. MSX2 promotes osteogenesis and suppresses adipogenic differentiation of multipotent mesenchymal progenitors. J Biol Chem. 2003;278:45969–45977. doi: 10.1074/jbc.M306972200 12925529 10.1074/jbc.M306972200

[55] Simionescu A Simionescu DT Vyavahare NR. Osteogenic responses in fibroblasts activated by elastin degradation products and transforming growth factor-beta1: role of myofibroblasts in vascular calcification. Am J Pathol. 2007;171:116–123. doi: 10.2353/ajpath.2007.060930 17591959 10.2353/ajpath.2007.060930 PMC1941602

[56] Canfield AE Doherty MJ Kelly V Newman B Farrington C Grant ME Boot-Handford RP. Matrix Gla protein is differentially expressed during the deposition of a calcified matrix by vascular pericytes. FEBS Lett. 2000;487:267–271. doi: 10.1016/s0014-5793(00)02363-2 11150522 10.1016/s0014-5793(00)02363-2

[57] Nakisli S Lagares A Nielsen CM Cuervo H. Pericytes and vascular smooth muscle cells in central nervous system arteriovenous malformations. Front Physiol. 2023;14:1210563. doi: 10.3389/fphys.2023.1210563 37601628 10.3389/fphys.2023.1210563 PMC10437819

