# Supplementary material for: Tuning BMP-Regulated Cell Differentiation in the Aortic Media by Mutating Matrix Gla Protein
Source: Arterioscler Thromb Vasc Biol. 2026 Jul 16;46(9):e324621. doi: 10.1161/ATVBAHA.126.324621 (PMC13506195; doi:10.1161/ATVBAHA.126.324621)
Supplement: Supplementary file 1 [file atv-46-e324621-s001.pdf]

SUPPLEMENTAL FIGURES and MAJOR RESOURCES TABLE

Enhanced Aortic Fibrosis but No Calcification after Mutation of BMP-Regulatory Element in Matrix Gla Protein

Xinjiang Cai, MD, PhD,[1,*] Kavinda K.J. Gunasinghe,[2,3] Lei Qi, MD, PhD,[1] Xiuju Wu, MD, PhD,[1] Li Zhang, PhD,[1] Jing Zheng, PhD,[1] Yan Zhao, PhD,[1] Hannah Kim,[1] Tzung Hsiai, MD, PhD,[1,4,5] Taufiq Rahman,[6] Xavier W. Chee,[2,7] Yucheng Yao, MD, PhD,[1] Kristina I. Boström, MD, PhD[1,8*]

[1] Division of Cardiology, Department of Medicine, David Geffen School of Medicine at University of California, Los Angeles, Los Angeles, CA, 90095, USA.
[2] Faculty of Engineering, Computing and Science, Swinburne University of Technology Sarawak, Kuching, Sarawak 93350, Malaysia.
[3] Medicinal Chemistry, Monash Institute of Pharmaceutical Sciences, Monash University, VIC 3052, Australia
[4] Department of Bioengineering, Henry Samueli School of Engineering and Applied Science, University of California Los Angeles, Los Angeles, CA 90095, USA.
[5] Division of Cardiology, Department of Medicine, Greater Los Angeles VA Healthcare System, Los Angeles, CA 90073, USA.
[6] Department of Pharmacology, University of Cambridge, Tennis Court Road, Cambridge CB2 1PD, UK.
[7] Department of Biochemistry, Yong Loo Lin School of Medicine, National University of Singapore, Singapore 117596, Singapore
[8] Molecular Biology Institute, University of California, Los Angeles, Los Angeles, CA, 90095, USA.

Short title: Vascular integrity and matrix Gla protein

Address correspondence to: Xinjiang Cai, MD, PhD, or Kristina Boström, MD, PhD, University of California, Los Angeles, Box 951679, Los Angeles, CA 90095-1679
Email: xinjiangcai@mednet.ucla.edu or kbostrom@mednet.ucla.edu

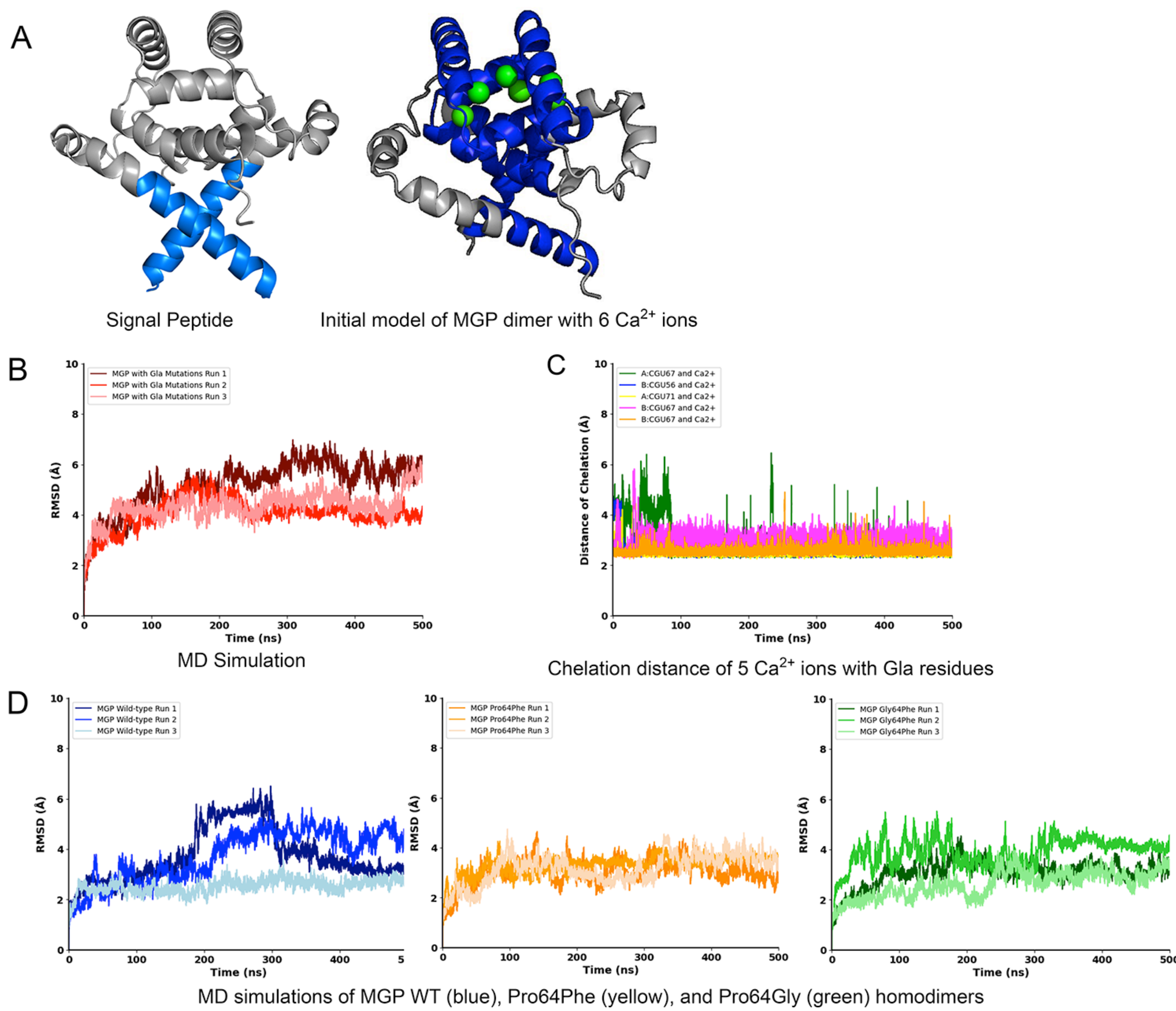

Figure S1. Working models of MGP dimer and Molecular Dynamics Simulation Studies.

A, MGP dimeric structure predicted by AlphaFold3. *Left*, light blue indicating the signal peptides of MGP. The signal peptide, which is absent in the mature protein, is removed in molecular dynamics simulations. *Right*, dark blue indicating the regions with high confidence pIDDT values >70.
B, Molecular dynamics simulations with γ-carboxylated Glu residues for 500 ns in triplicates. The RMSD stabilized around the 300 ns-mark with slight fluctuation (< 2 Å) in replicate 3 at around the 300 ns-mark.
C, The chelation distance between each Ca2+ ion and their potential binding Gla residues based on the most stable conformation in replicate 2. In all cases, the average distance remains less that 3 Å.
D, Molecular dynamics simulation studies and protein RMSD analysis of MGP WT (left), Pro64Phe (middle), and Pro64Gly (right) for 500 ns in triplicate.

Abbreviations: pIDDT, predicted Intrinsic Disorder and Template Discrepancy Tolerance; MGP, Matrix Gla Protein; RMSD, Root Mean Square Deviation.

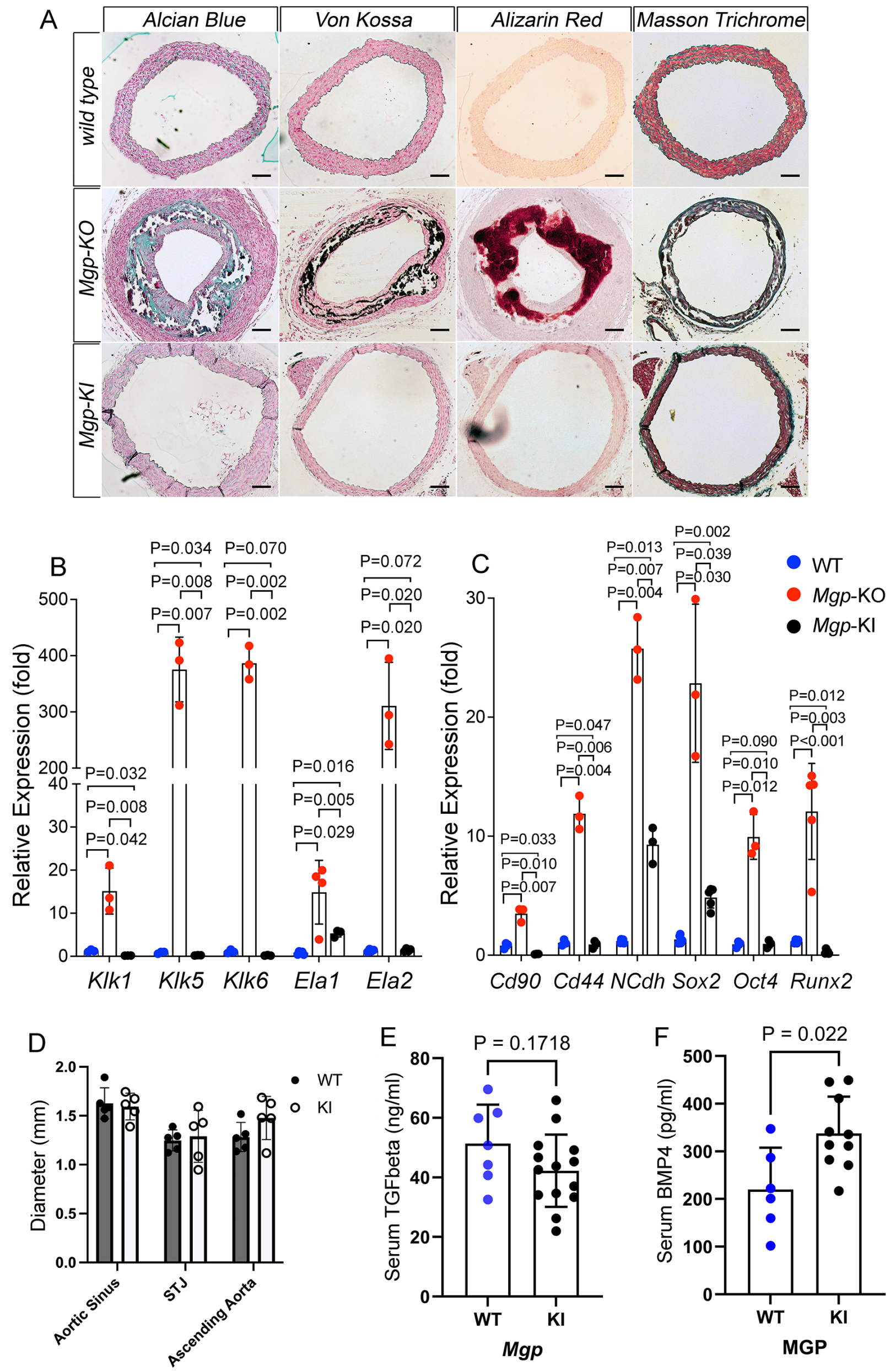

Figure S2. Histochemical staining and gene expression of Mgp-Knockin (KI) aorta.

A. Histochemical staining (Alcian Blue, Von Kossa, Alizarin Red and Masson Trichrome) of aorta from wild-type, *Mgp*-KO and *Mgp*-KI mice at 4 weeks of age. Scale bars, 50 µm. Representative of 3 independent experiments (sections from 2 males, 1 female).
B and C. *Left*, aortic expression of the proteases Kallikrein 1, 5 and 6 (*Klk1, Klk5, Klk6*) and Elastase 1 and 2 (*Ela1, Ela2*) in wild-type (WT), *Mgp*-KO and *Mgp*-KI mice at 4 weeks of age, as determined by qPCR. *Right*, Aortic expression of markers for endothelial-mesenchymal transitions (EndMTs) (*Cd90, Cd44, N-Cadherin, Sox2, Oct4*) and the osteogenic marker Runx2 in wild-type (WT), *Mgp*-KO and *Mgp*-KI mice at 4 weeks of age, as determined by qPCR (n=3 mice per group; 2 males, 1 female). One-way ANOVA with Tukey post hoc test.
D. Echocardiographic measurements of sizes of aortic sinus, sinotubular junction (*STJ*) and middle ascending aorta in WT and *Mgp*-KI mice (n=5 mice per group; 5 males).
E. Total serum TGFβ concentration in WT (7 mice; 4 males 3 females) and *Mgp*-KI (14 mice; 7 males, 7 females). Unpaired two-tailed Student's t tests.
F. Total serum BMP4 concentration in WT (6 mice; 3 males 3 females) and *Mgp*-KI (10 mice; 5 males, 5 females). Unpaired two-tailed Student's t tests.

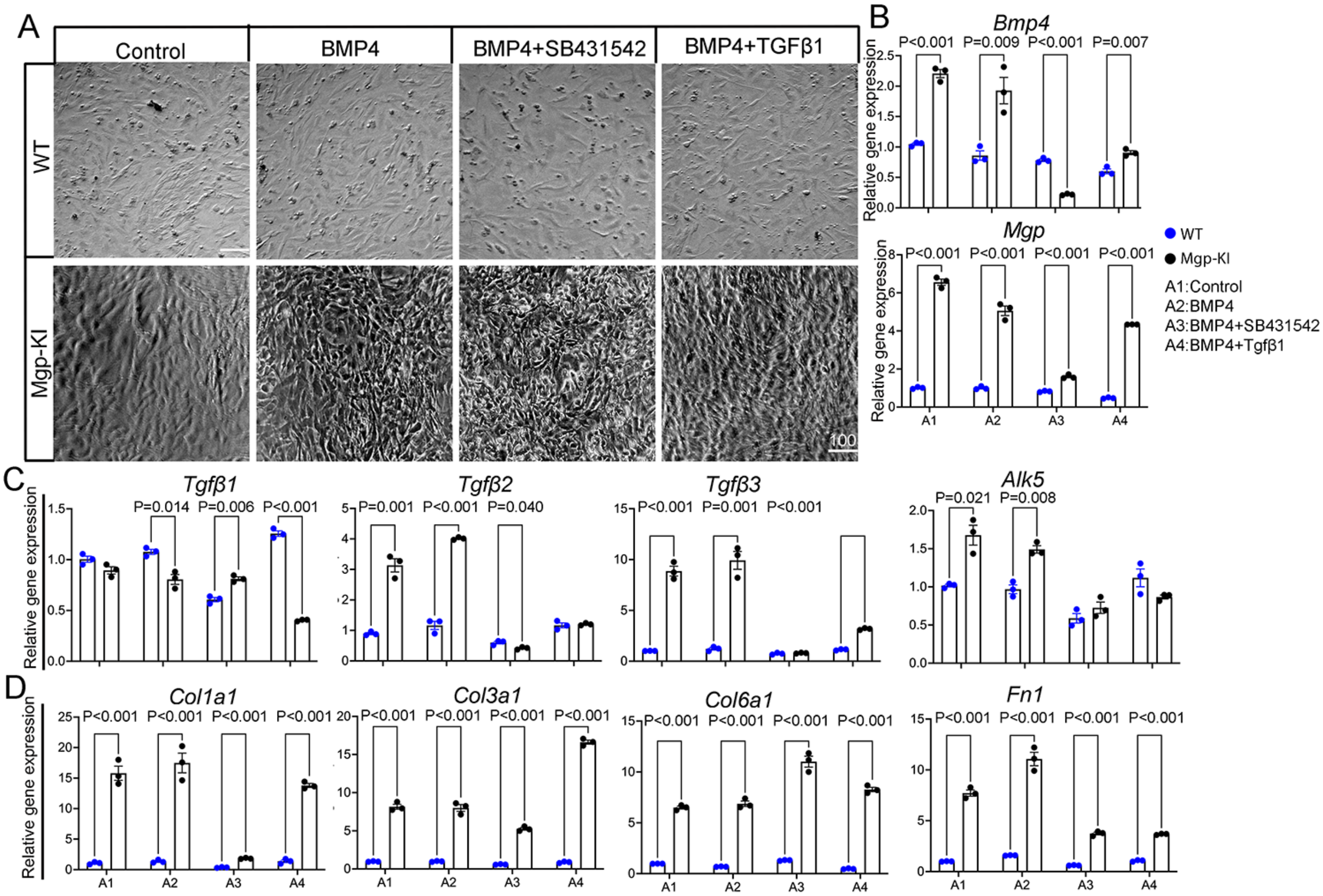

Figure S3. Effect of BMP and TGFβ signaling on marker gene expression in aortic cells isolated from wild-type (WT) and *Mgp*-KO mice.

Cells were treated with BMP4 (50 ng/ml), either alone or in combination with the TGFβ inhibitor SB431542 (5 μmol/l) or TGFβ1 (5 ng/ml) for 48 hours. Aortic cells were prepared from WT mice (4 males, 4 females) and *Mgp*-KI mice (3 males, 3 females).
(A) Representative images of WT and *Mgp*-KI cells with and without treatments.
Expression of (**B**) *Bmp4* and *Mgp*, (**C**) TGFβ components (*Tgfb1, Tgfb2, Tgfb3* and *Alk5*), (**D**) Extracellular matrix genes (*Col1a1, Col3a1, Col6a1* and *Fn1*) as determined by qPCR (n=3 mice per group; 2 males, 1 female). One-way ANOVA with Tukey post hoc test.

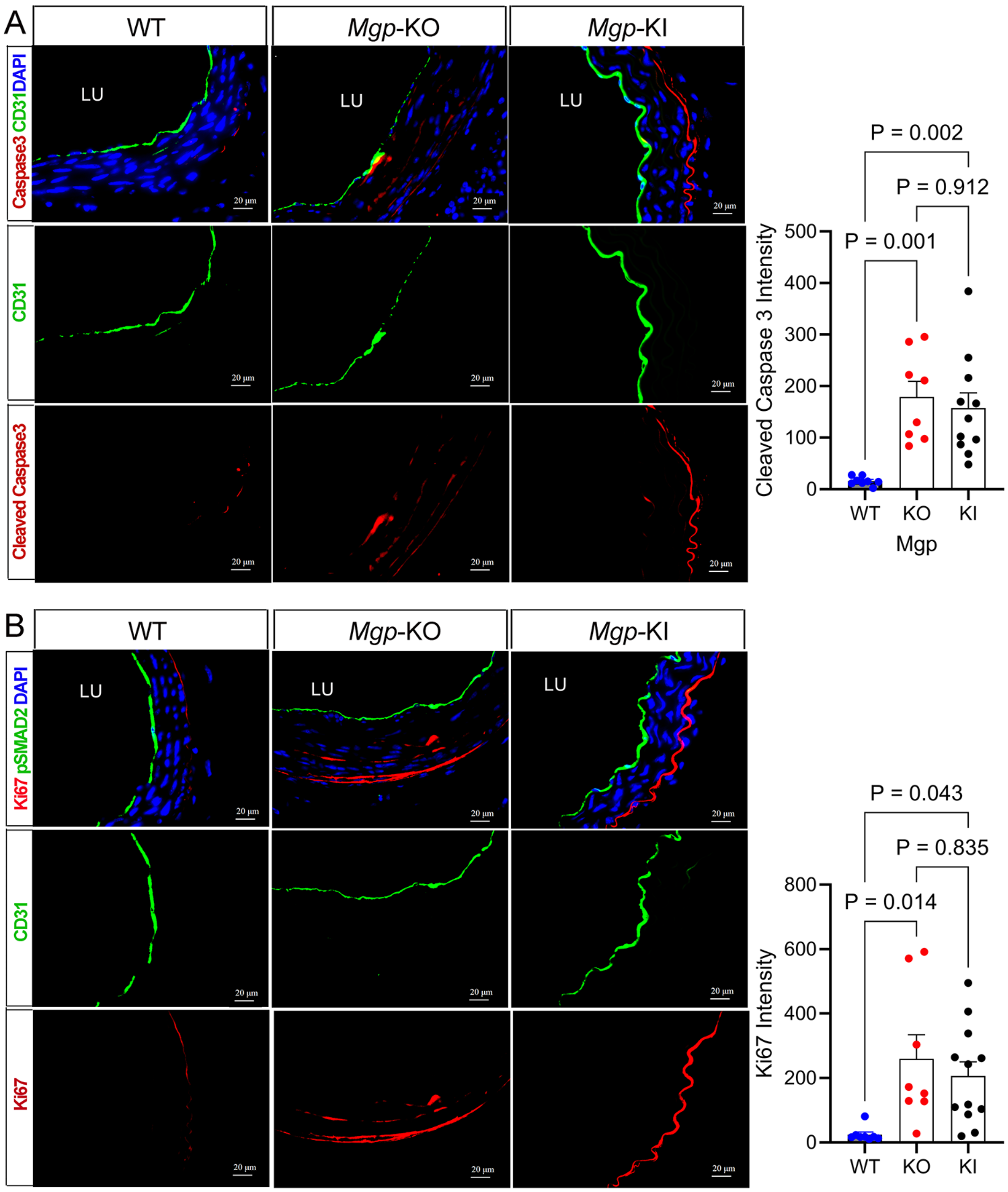

Figure S4. Immunofluorescence for (A) cleaved caspase-3, and (B) Ki67 in aortas from wild-type (WT), *Mgp*-KO and *Mgp*-KI mice.

DAPI was used to visualize nuclei. Bars, 20 µm. Intensity determined by Image J (n=3 sections from male mice; 3 fields of view per section). One-way ANOVA with Tukey post hoc test. Data are shown as mean ± SEM.

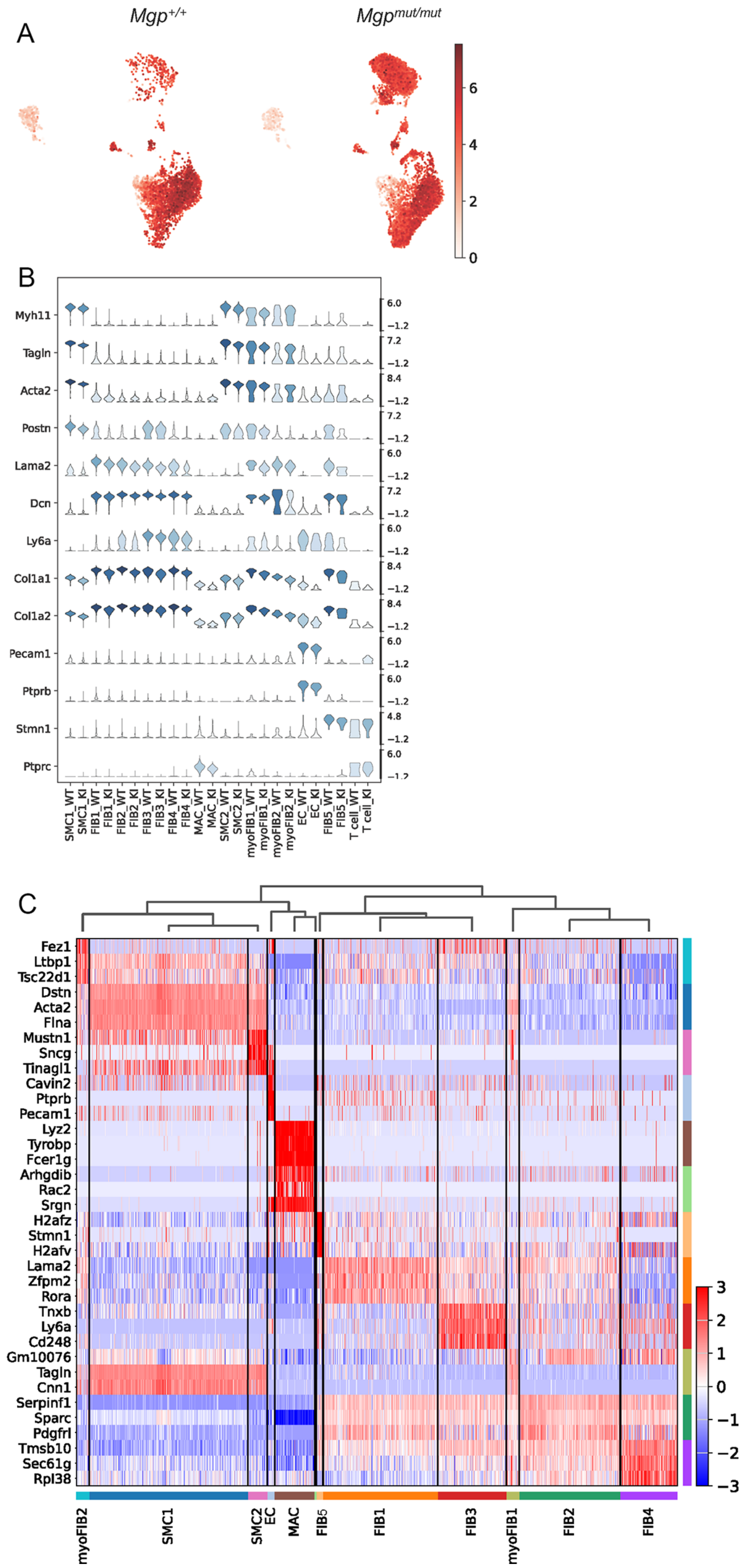

Figure S5. Comparison of gene expression levels of *Mgp* (A), violin plot (B) and heat map (C) of top marker genes for different cell types.

Abbreviations: *EC*, endothelial cells; *FIB*, fibroblasts; *MAC*, macrophages; *myoFIB*, myofibroblasts; *SMC*, smooth muscle cells.

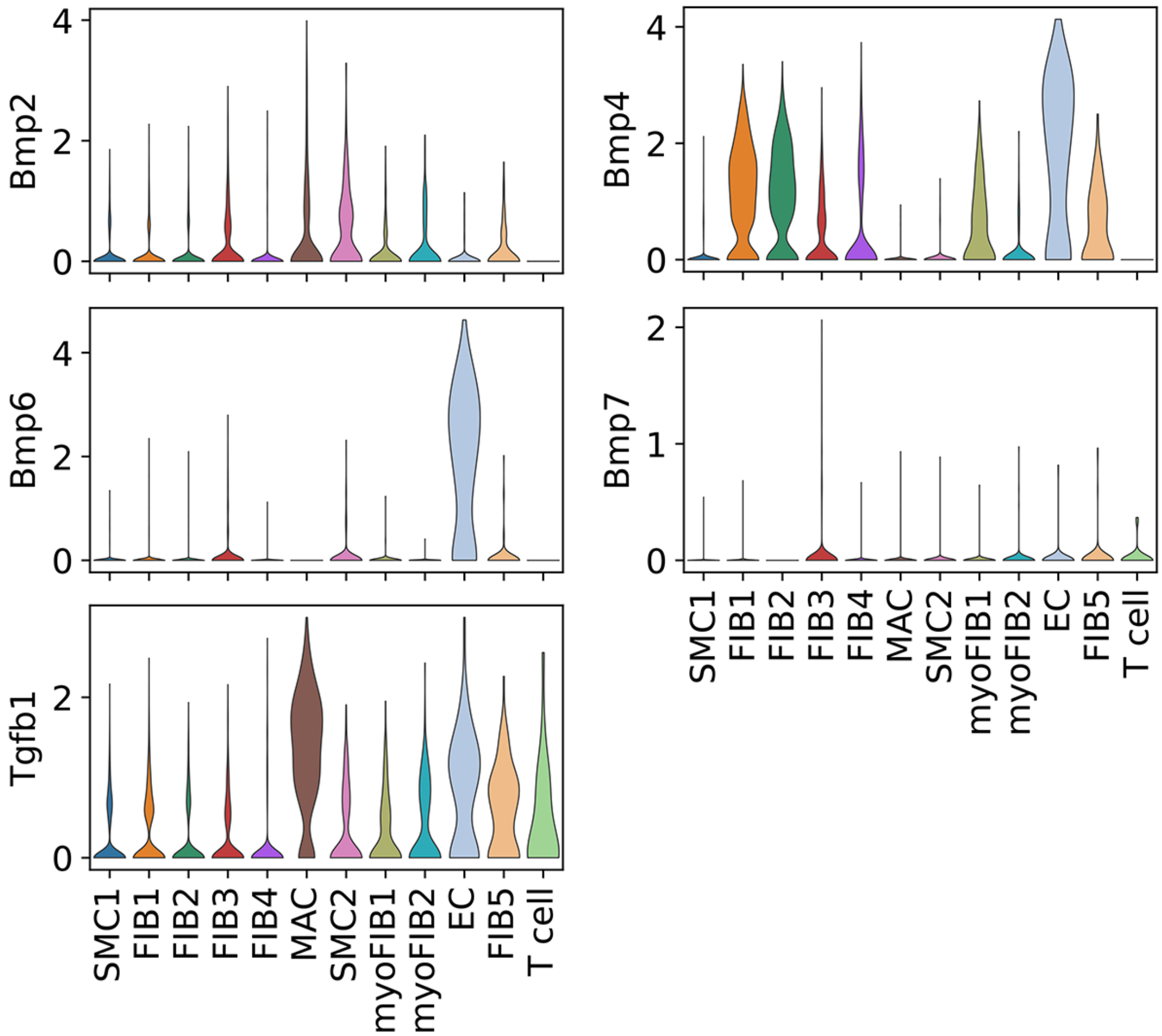

Figure S6. VInplots of *Bmp2*, *Bmp4*, *Bmp6*, *Bmp7* and *Tgfb1* in aortic cell populations from single cells-RNA sequencing.

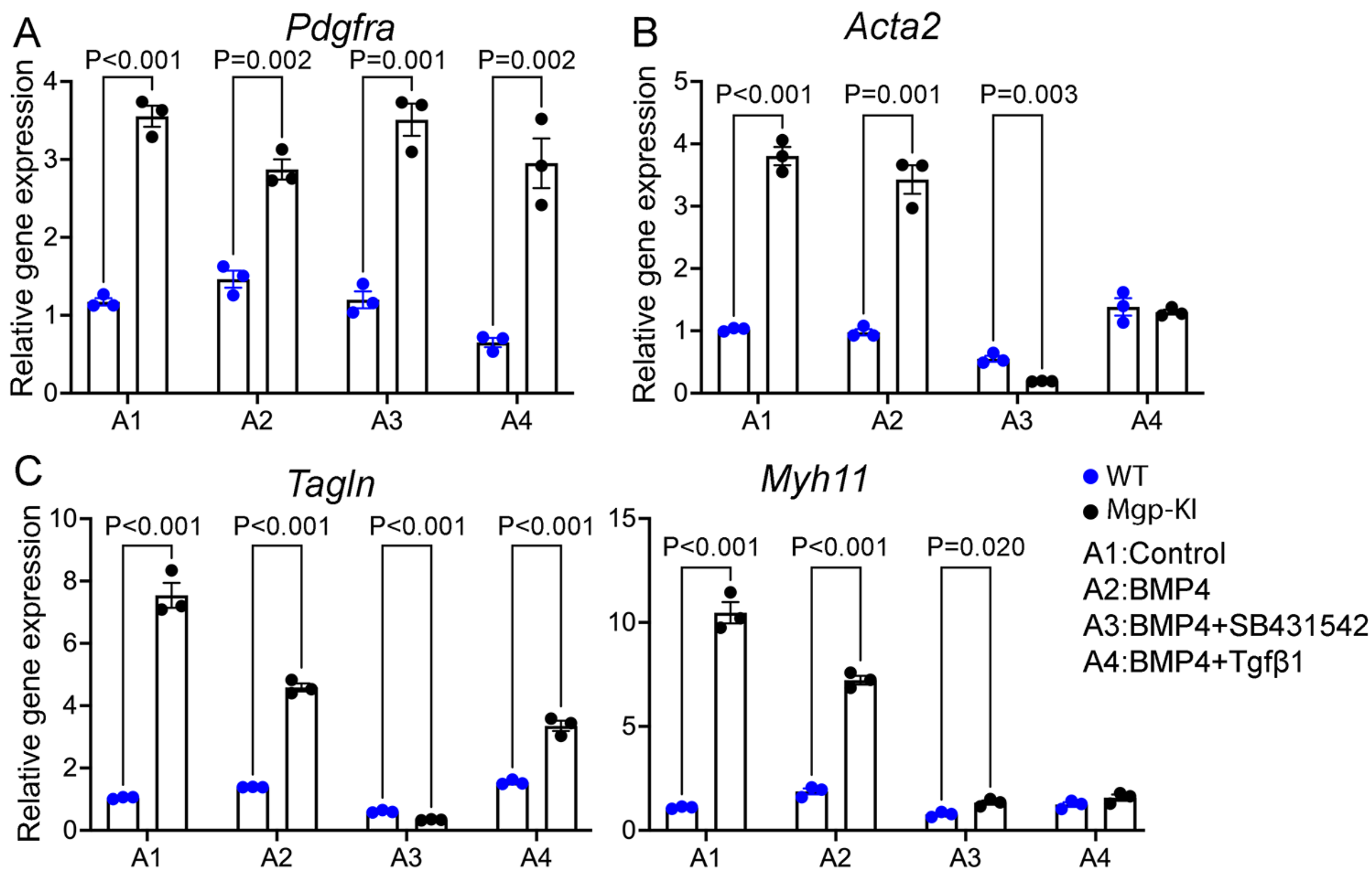

Figure S7. Expression of marker genes in aortic cells isolated from wild-type (WT) and *Mgp*-KO mice.

(A) *Pdgfra*, (**B**) *Acta2,* (**C**) *Tagln* and *Myh11,* as determined by qPCR (n=3 mice per group; 2 males, 1 female). One-way ANOVA with Tukey post hoc test.

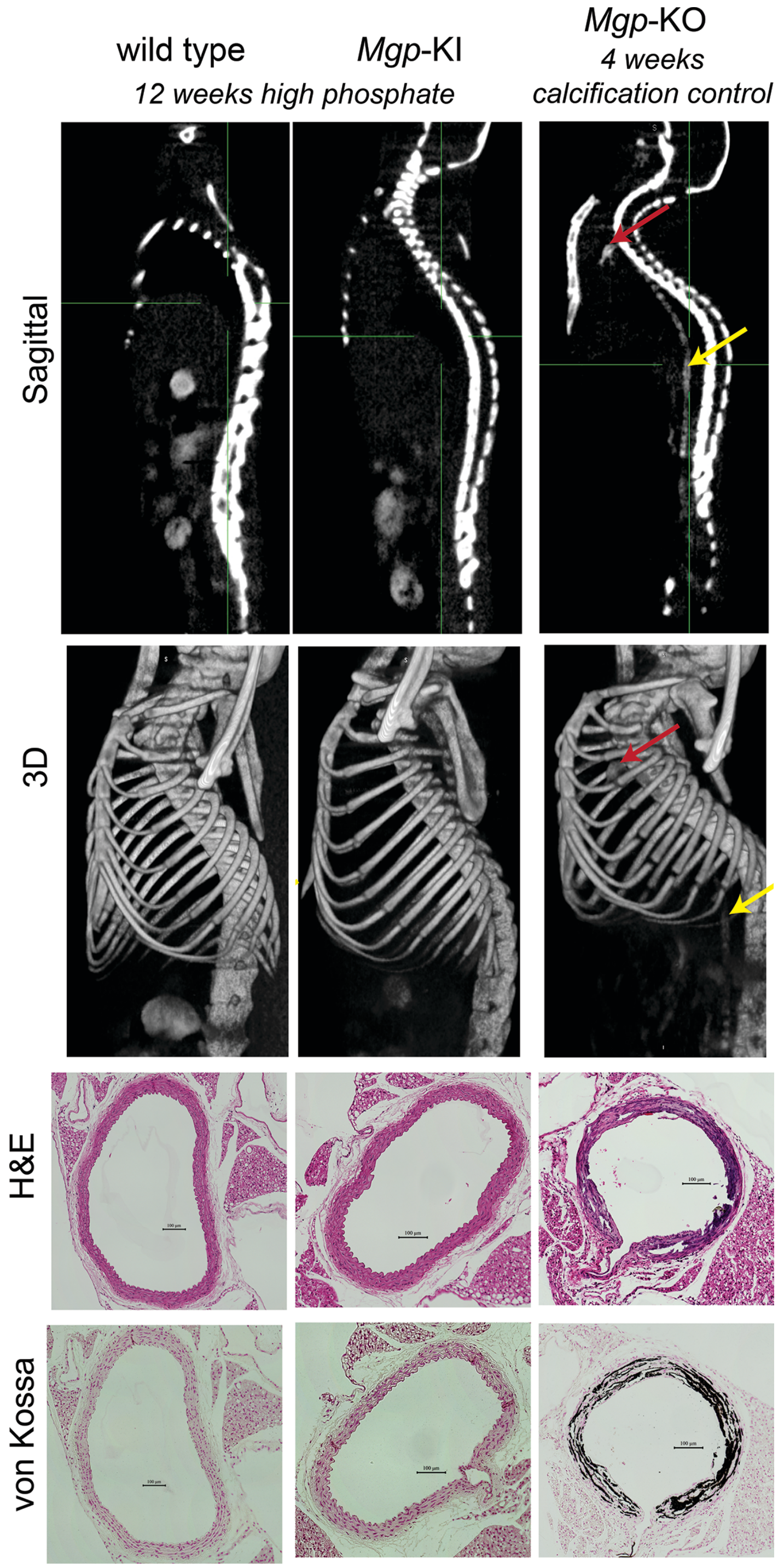

Figure S8. High phosphate diet did not induce vascular calcification in *Mgp*-KI mice.

A, MicroCT imaging of sagittal and 3D-convolution of wild-type (WT) and Mgp-KI mice after being fed for 12 weeks of high phosphate diet starting at 4-week-old. *Arrows* indicate aortic calcification in control 4-week-old *Mgp*-KO mice.

B, Histochemical staining (H&E and Von Kossa,) of aorta from wild-type, *Mgp*-KI, and control *Mgp*-KO mice. Scale bars,100 μm (representative of 4 replicate experiments; 2 males, 2 females).

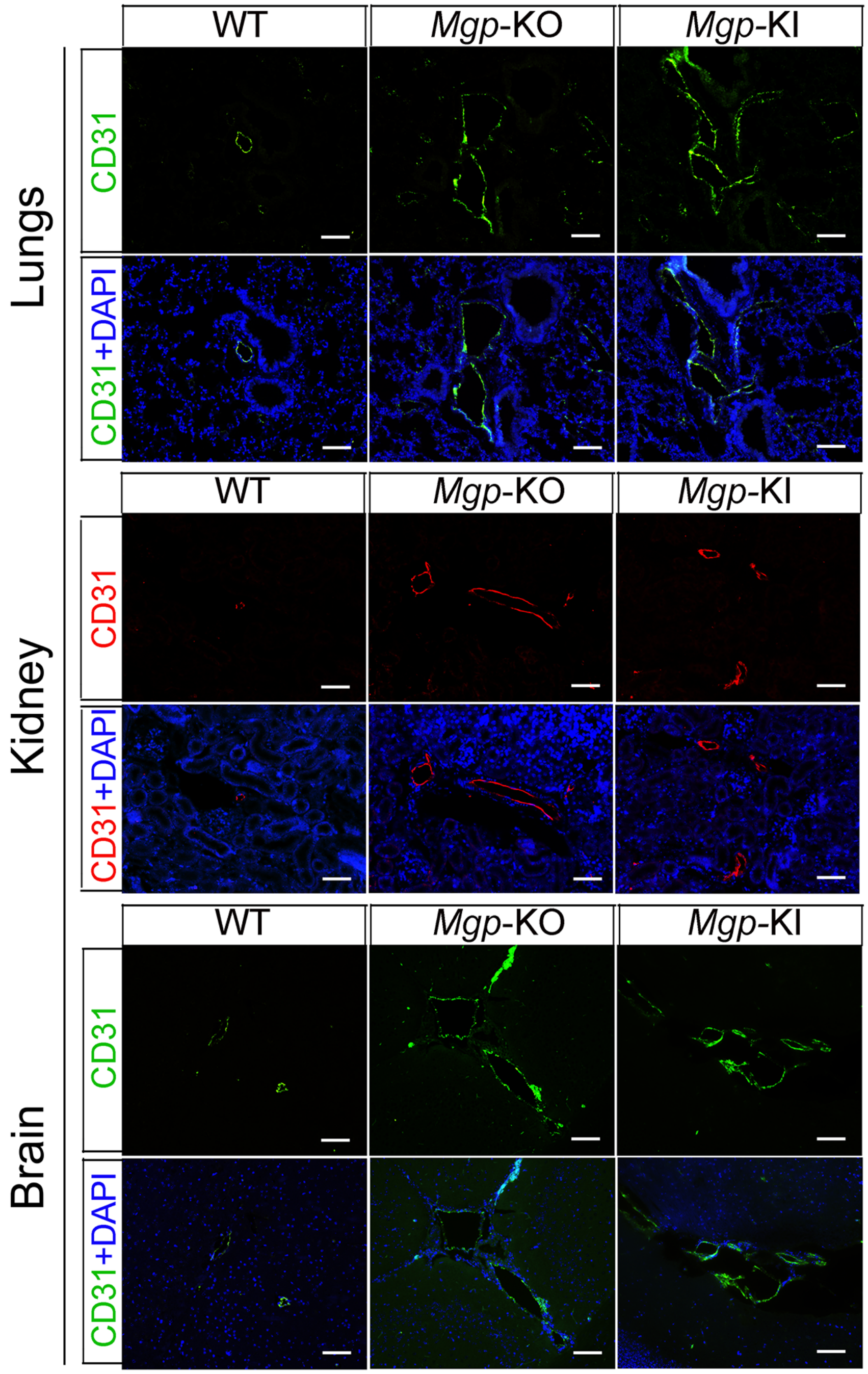

Figure S9. Vascular malformations in multiple organs in *Mgp*-KI mouse.

Immunofluorescence for the endothelial marker CD31 (green, panels for lungs and brain), CD31 (red, panel for kidneys) of vasculature in lungs, kidneys and brain from wild-type (WT), *Mgp*-KO and *Mgp*-KI mice. DAPI was used to visualize nuclei. Bars, 100 µm (representative of 4 replicate experiments; 2 males, 2 females).

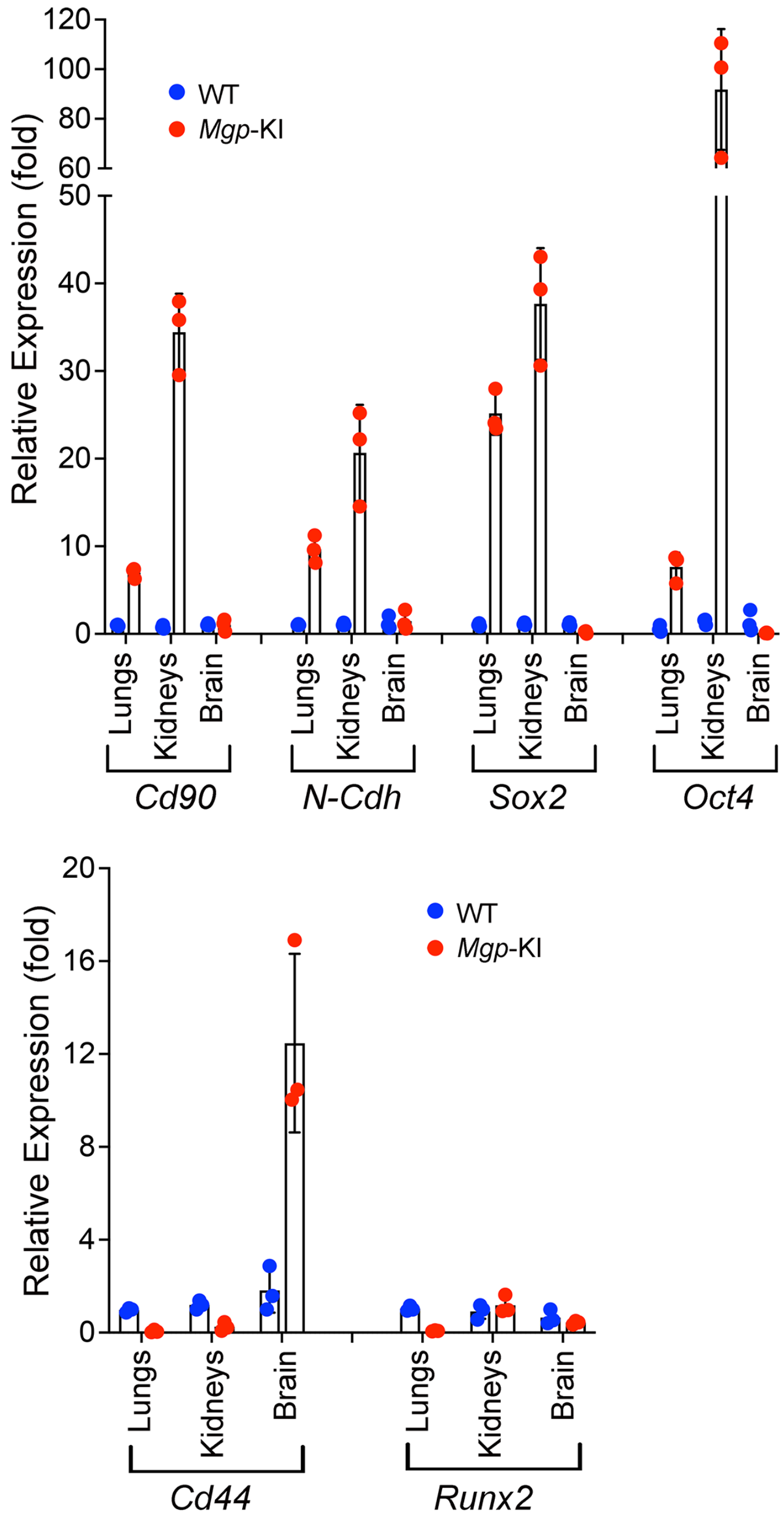

Figure S10. Marker gene expression in lungs, kidneys, and brain between WT and *Mgp*-KI mice .

Comparison of expression of markers for EndMTs (*Cd90, N-Cadherin, Sox2, Oct4, Cd44*) and osteogenesis (*Runx2*) in lungs, kidneys and brain from wild-type (WT) and *Mgp*-KI mice at 4 weeks of age, as determined by qPCR (n=3 mice per group; 2 males, 1 female).

Major Resources Table

Animals (in vivo studies)

Species	Vendor or Source	Background Strain	Sex	Persistent ID / URL
Mouse	Jackson Laboratory and Dr. Kristina Bostrom's lab	C57BL/6J	M&F	

Genetically Modified Animals

	Species	Vendor or Source	Background Strain	Other Information	Persistent ID / URL
**Parent - Male**	Mouse	Jackson Laboratory	C57BL/6J	$Mgp^{+/-}$	Stock No: 023811
**Parent - Female**	Mouse	Jackson Laboratory	C57BL/6J	$Mgp^{+/-}$	Stock No: 023811
**Parent - Male**	Mouse	Dr. Kristina Bostrom's lab	C57BL/6J	$Mgp^{mut/mut}$	MGP Pro64Gly mutation
**Parent - Female**	Mouse	Dr. Kristina Bostrom's lab	C57BL/6J	$Mgp^{mut/mut}$	MGP Pro64Gly mutation

Antibodies

Target antigen	Vendor or Source	Catalog #	Working concentration	Lot # (preferred but not required)	Persistent ID / URL
CD31, goat polyclonal	R&D systems	AF3628	1:200		
MGP, rabbit polyclonal	Abcam	ab86233	1:2,000		
Alpha-smooth muscle actin, mouse monoclonal	R&D Systems	MAB1420	1:200		
Phospho-SMAD2 rabbit monoclonal	Cell Signaling Technology	18338	1:200		
phospho-SMAD1/5/8, rabbit	Millipore Sigma	AB3848-I	1:200		
vWF, rabbit polyclonal	Dako	A0082	1:200		
Ki-67, Rabbit Monoclonal (SP6)	ThermoFisher	MA5-14520	1:200		
Cleaved Caspase-3 (Asp175), Rabbit polyclonal	Cell Signaling Technology	9661	1:200		
4',6-diamidino-2-phenylindole (DAPI)	Sigma-Aldrich	D9542	1:5000		

DNA/cDNA Clones

Clone Name	Sequence	Source / Repository	Persistent ID / URL
N/A			

Cultured Cells

DOI [to be added]

Name	Vendor or Source	Sex (F, M, or unknown)	Persistent ID / URL
N/A			

Data & Code Availability

Description	Source / Repository	Persistent ID / URL
scRNA sequencing	NCBI GEO	Accession number GSE289219
Seurat v5		https://satijalab.org/seurat/
Monocle3		https://github.com/cole-trapnell-lab/monocle3/blob/master/NEWS.md

Other

Description	Source / Repository	Persistent ID / URL
High phosphate diet (2% P & 1.2% Ca)	Inotiv	Cat no. TD.120538
RNeasy Mini Kit	Qiagen	Cat# 74106
High-Capacity cDNA Reverse Transcription Kit	Thermo Fisher	Cat# 4374967
TaqMan™ Universal Master Mix II, With UNG	Thermo Fisher	Cat# 4440039
MicroView 3-D volume viewer and analysis tool software	GE Healthcare	
Osirix software	Pixmeo SARL	
GFP-labeled fluorescent microsphere	FluoSpheres™, Invitrogen	F21010
Mouse TGF-beta 1 DuoSet ELISA	R&D systems	DY1679

Primer	Oligonucleotides / Cat#	Experiment
Gapdh	Mm99999915_g1 (Thermo Fisher Scientific)	RT-qPCR
Mgp	Mm01615497_m1	RT-qPCR
Bmp2	Mm01340178_m1	RT-qPCR
Bmp4	Mm00432087_m1	RT-qPCR
Alk1	Mm00437432_m1	RT-qPCR
Alk2	Mm01331069_m1	RT-qPCR
Alk5	Mm00436964_m1	RT-qPCR
Tgfb1	Mm01178820_m1	RT-qPCR
aSMA	Mm00725412_s1	RT-qPCR
Mgp_WT-F	GCCACAATTTCTGCATCCTGC	Genotyping
Mgp_WT-R	CGGGAAAGATGAGGAAGAAGGG	Genotyping
Mgp-KO-F	TGCCTGAAGTAGCGGTTGTA	Genotyping
Mgp-KO-R	TGAATGAACTGCAGGACGAGG	Genotyping
Mgp-KI-1F	GGCTCCATGCTTAAGAGCAC	Genotyping
Mgp-KI-1R	GCTTGGCTGGACGTAAACTCCT	Genotyping
Mgp-KI-2R	CTCCTGTGTCTGACCCCAAT	Genotyping

DOI [to be added]

ARRIVE GUIDELINES

The ARRIVE guidelines (https://arriveguidelines.org/) are a checklist of recommendations to improve the reporting of research involving animals. Key elements of the study design should be included below to better enable readers to scrutinize the research adequately, evaluate its methodological rigor, and reproduce the methods or findings.

Study Design

Groups	Sex	Age	Number (prior to experiment)	Number (after termination)	Littermates (Yes/No)	Other description
Wild-type	M&F	4 weeks	3-7	3-7	No	For Histology, RT-qPCR
Mgp-KI	M&F	4 weeks	3-7	3-7	No	For Histology, RT-qPCR
Mgp-KO	M&F	4 weeks	3-7	3-7	No	For Histology, RT-qPCR
Wild-type	M&F	4 weeks	10	10	No	For scRNA
Mgp-KI	M&F	4 weeks	10	10	No	For scRNA

Sample Size:

The sample size was determined based on prior experiments with wild-type and genetically engineered mice. Where applicable, sample size calculations were performed to achieve 80% power and a 0.05 significance level (Type I error). Group sizes were informed by variability in previous studies with a balance in statistical rigor with practical feasibility.

Inclusion Criteria

Healthy 4-week-old mice

Exclusion Criteria

None.

Randomization

None.

Blinding

Histology samples were assessed by independent investigators in a blind manner.

DOI [to be added]
